# Partial restoration of gut‐mucosal dysbiosis in late‐treated HIV‐infected subjects with CD4 T‐cell recovery

**DOI:** 10.1002/ctm2.788

**Published:** 2022-04-05

**Authors:** Israel Olivas‐Martínez, Isaac Rosado‐Sánchez, Juan Antonio Cordero‐Varela, Salvador Sobrino, Miguel Genebat, Inés Herrero‐Fernández, Rocío Martínez de Pablos, Ana Eloísa Carvajal, Rocío Ruiz, Ana Isabel Álvarez‐Ríos, María Fontillón‐Alberdi, Ángel Bulnes‐Ramos, Vanesa Garrido‐Rodríguez, María del Mar Pozo‐Balado, Manuel Leal, Yolanda María Pacheco

**Affiliations:** ^1^ Immunology Lab, Institute of Biomedicine of Seville University Hospital Virgen del Rocío/CSIC/University of Seville Seville Spain; ^2^ Bioinformatics and Computational Biology Service, Institute of Biomedicine of Seville University Hospital Virgen del Rocío/CSIC/University of Seville Seville Spain; ^3^ Digestive Service University Hospital Virgen del Rocío Seville Spain; ^4^ Biochemistry and Molecular Biology Department, Pharmacy, University of Seville, Spain; Institute of Biomedicine of Seville University Hospital Virgen del Rocío/CSIC/University of Seville Seville Spain; ^5^ Biochemistry Service University Hospital Virgen del Rocío Seville Spain; ^6^ Service of Pathological Anatomy University Hospital Virgen del Rocío Seville Spain; ^7^ Internal Medicine Service Viamed‐Santa Ángela Hospital Seville Spain

Dear Editor,

Dysbiosis of the gut microbiome is commonly found in human immunodeficiency virus (HIV)‐infected subjects,^1^ related to alterations such as microbial translocation, inflammation and chronic immune activation.^2^ These alterations especially affect late‐diagnosed and/or late‐treated individuals, who represent 40%–60% of HIV‐subjects and have higher morbimortality.^3^ Furthermore, a quarter of the late‐treated subjects do not properly recover CD4 T‐cell levels after combined antiretroviral therapy (cART) and suffer an even worse clinical evolution (see Supporting Information‐text for extended context).^3^ There is no consensus on a single sample type or gut location suitable for studying dysbiosis, but reports mostly rely on faecal samples.^4^, ^5^ However, interactions with the HIV‐targeted gut‐associated lymphoid tissue mainly depend on mucosal‐specific microorganisms.^6^ Moreover, studies comparing alterations in different intestinal sites are scarce.^7^ Additionally, most studies have explored the effect of infection itself^8^ or from cART^9^ on the gut microbiota. However, potential associations between immune status and changes occurring in the gut microbiome of treated HIV‐infected subjects have not been explored yet.

Our hypothesis was that clinical phenotypes with poorer immune status (in terms of CD4 counts before and after cART) would present a more dysbiotic microbiome in their gut mucosa. Thus, we aimed to explore potential differences in specific dysbiosis in the gut‐mucosal microbiome, comparing biopsies from two different locations (ileum and caecum), of HIV‐infected subjects with different clinical phenotypes. Associations with parameters of inflammation, immune activation and gut tissue damage were also explored.

Biopsies of terminal ileum and caecum mucosa, as well as blood samples, were taken from 35 virologically‐suppressed HIV‐infected subjects after at least a 2‐year cART period during colonoscopies performed at Virgen del Rocío University Hospital between 2014 and 2017. These patients were classified into three groups depending on their CD4 levels at cART onset and their response upon receiving therapy, according to Figure 1. Ten healthy (non‐HIV) people, as control subjects, and three elite controllers (ECs) were also enrolled (description of groups available at Table S3).

**FIGURE 1:**
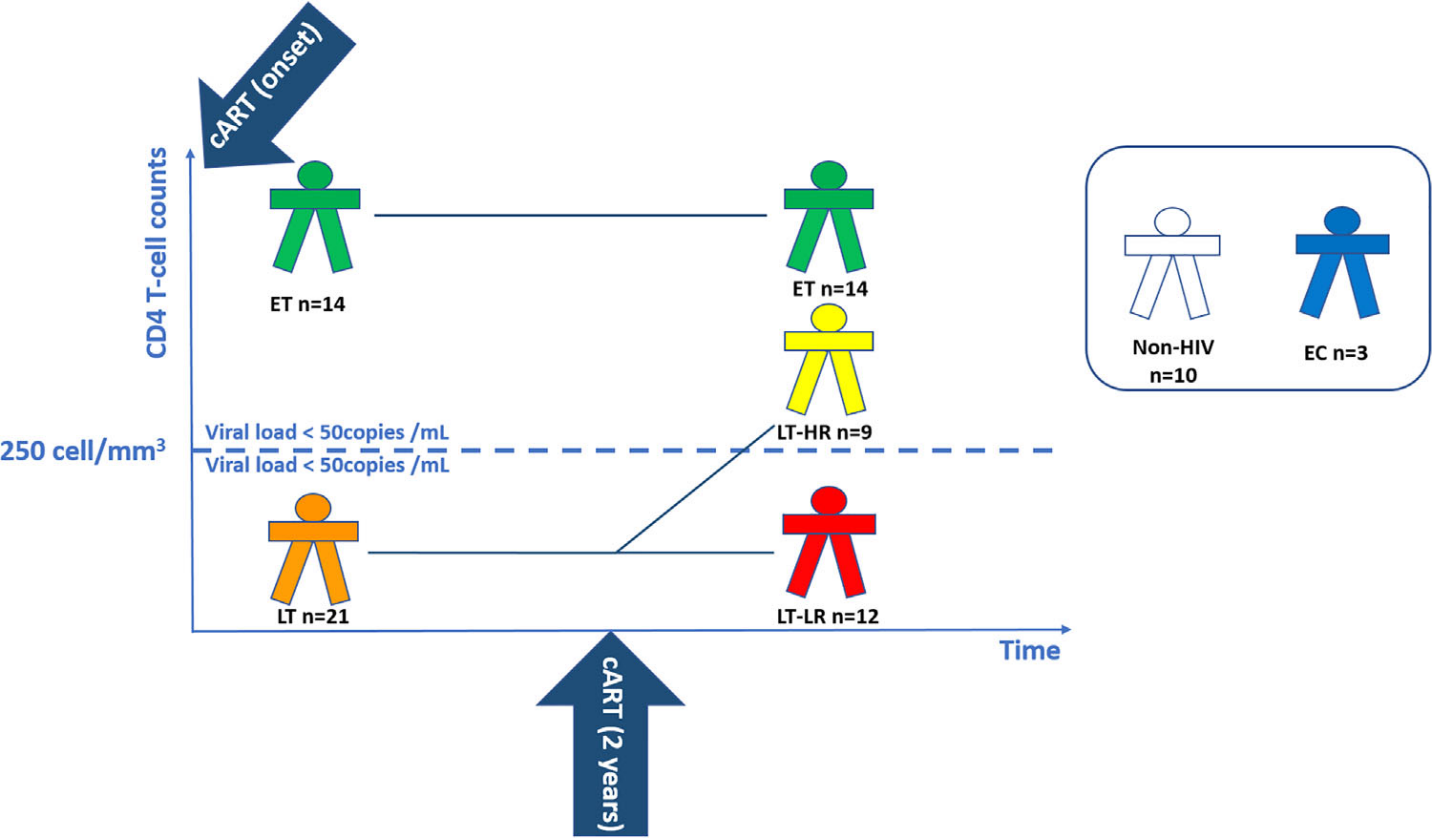
Schematic diagram showing the selection criteria and number of subjects enrolled in each study group. The following human immunodeficiency virus (HIV)‐treated groups (ET, LT‐HR and LT‐LR) were recruited according to immunological criteria: early‐treated (ET, *n* = 14), who started cART with >250 CD4 T cells/mm^3^ and remained >250 CD4 T cells/mm^3^ 2 years later; late‐treated high recovery (LT‐HR, *n* = 9), starting last cART with <250 CD4 T cells/mm^3^ but reaching >250 CD4 T cells/mm^3^ 2 years later; late‐treated low‐recovery (LT‐LR, *n* = 12), starting cART with <250 CD4 T cells/mm^3^ and remaining <250 CD4 T cells/mm^3^ 2 years later. All these cART‐treated HIV‐infected subjects were virologically suppressed (<50 HIV RNA copies/ml). Additionally, a reduced group of elite controllers (EC, *n* = 3) was also included. EC are HIV‐subjects with spontaneous virological suppression in the absence of cART. Control healthy subjects (*n* = 10) were individuals with similar age and sex characteristics to those of treated HIV‐subjects. Blood and biopsy samples of ileum and caecum mucosa were taken during colonoscopy procedures made a variable time after compliance of 2 years of cART as the classification period. All subjects maintained their classification criteria at the moment of collection of samples. Exclusion criteria for this study were having HIV rebounds during the classification period and until the recruitment; intestinal infections, cancer, active hepatitis C virus (HCV) infection or concurrent inflammatory processes at the moment of recruitment. All the subjects enrolled in this study were properly informed and signed the corresponding informed consents either for the colonoscopy and the experimental study. ET, early‐treated; LT, late‐treated; LT‐HR, late‐treated high recovery; LT‐LR, late‐treated low recovery; EC, elite controllers; cART, combined antiretroviral therapy

Sequencing data of 16S rRNA from the microbiota present in terminal ileum and caecum samples of study subjects were analysed and compared with bacterial taxonomic databases. Thus, parameters of richness and alpha‐diversity of the gut microbiome showed no statistically significant differences between healthy and HIV‐groups (elite controller [EC], early‐treated [ET], late‐treated high recovery [LT‐HR] and late‐treated low recovery [LT‐LR]) regardless the biopsied intestinal area (Figure 2A) or patient sex (Figure S1). Even so, healthy and EC groups showed higher mean values of richness and diversity than ET and LT HIV‐groups. A strong positive correlation was found (*r* > .8; *p* < .0001) when alpha‐diversity was compared between ileum and caecum samples of all study groups (Figure 2B). In addition, estimation of beta‐diversity through Non‐metric multidimensional scaling (NMDS) analysis revealed a clustering of healthy and EC samples compared to the rest of HIV‐groups (PERMANOVA, *p* < .001; HOMOVA, *p* < .041) (Figure 2C). These results were also significant and even clearer when distances were calculated by axis, especially in NMDS1 (Figure 2D). As for alpha diversity, beta diversity did not show relevant differences when comparing ileum and caecum samples (Figure S2). Thus, further abundance analyses were performed without separating by gut location.

FIGURE 2Richness and diversity measures of the microbiota obtained from gut mucosal samples of subjects included in the study groups. Sequencing data of microbial 16S rRNA were processed with Mothur (version 1.43.0), using SILVA (non‐redundant version 138) and Greengenes (version 13_8_99) databases for aligning and taxonomic purposes, respectively. Operational taxonomic units (OTUs) picking was performed at 97% sequence similarity to detect subgenera. Subsequent data analysis was accomplished using the Phyloseq package (R version 3.6.3). Only bacterial taxa present at least in 50% of the analysed samples were considered. (A) Different richness (Chao1 and ACE) and alpha diversity (Shannon and Simpson) indexes were calculated using Phyloseq and Vegan packages from R, and compared among study groups according to the location of sampling area for biopsies, caecum or ileum mucosa (dots represent outliers). (B) Spearman's Rho Coefficient was used to correlate Shannon and Simpson alpha diversity values between caecum and ileum samples of all the groups studied. (C) Non‐metric multidimensional scaling (NMDS) analysis for beta microbiome diversity of ileum and caecum samples from study groups’ subjects, choosing Morisita‐Horn index as it yielded the lowest stress value. Coloured dots represent samples from the different groups according to the legend on the right. (D) NMDS analysis separated by axes, NMDS1, and 2, showing distances among samples of the five study groups (dots represent outliers). Kruskal–Wallis rank sum tests, followed by post hoc Tukey's procedure, were used to test for differences in alpha diversity measures and NMDS axes among study groups. Differences in beta diversity were tested using PERMANOVA and HOMOVA procedures. *p* < .05 indicates a statistically significant association. EC, elite controllers; ET, early‐treated; LT‐HR, late‐treated high recovery; LT‐LR, late‐treated low recovery; r, Spearman's rho

**Figure:**
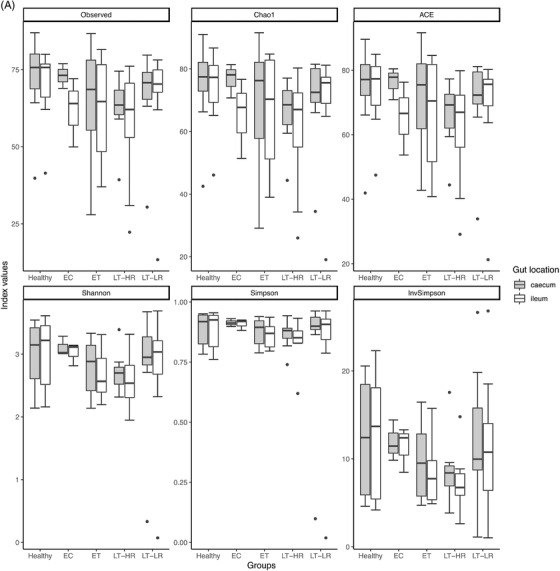

**Figure:**
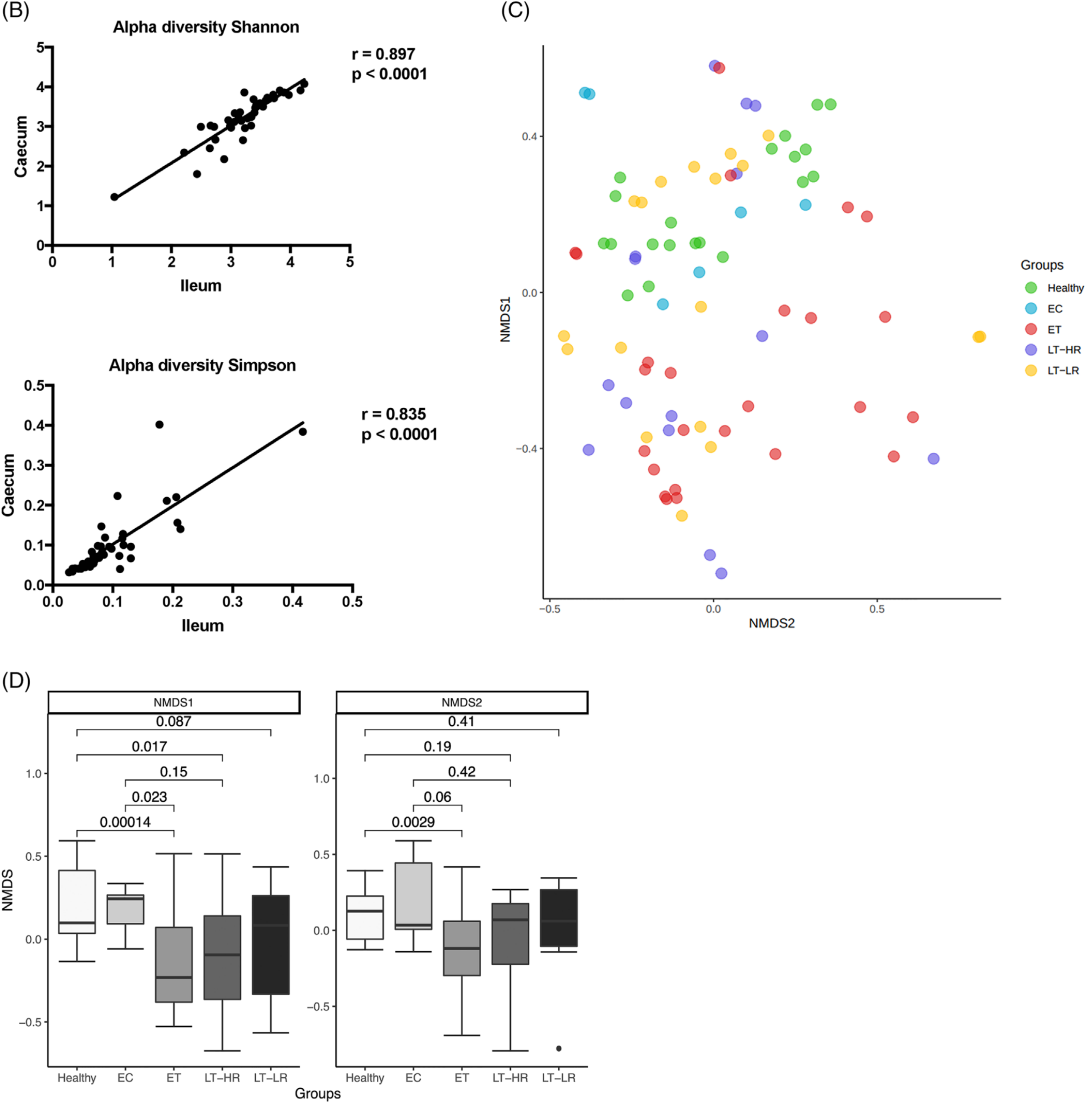

When operational taxonomic units (OTUs) identified in gut mucosal samples were compared between groups at phylum level, EC showed the highest relative abundance of Firmicutes (*p* ≤ .07), but lower abundance of Actinobacteria compared to ET (*p* = .04), LT‐HR (*p* = .006) and LT‐LR (*p* = .01) groups (Figure 3A). At class level, Clostridia, Bacteroidia and Deltaproteobacteria were more significantly abundant in healthy and EC groups (*p* ≤ .03) than treated HIV‐groups, who showed instead higher relative abundance of Bacilli, Erysipelotrichi, Actinobacteria or Coriobacteriia (*p* ≤ .03) (Figure S3). Using DESeq2 methodology, OTUs appearing as significantly different in relative abundance at family/genus level were compared among study groups (Figures 3B and S4A). For a visual simplification of all comparisons, profiles of taxonomic grouping of more abundant OTUs, in their respective genus and/or family taxa, in comparisons of each study group with the rest of groups was also performed (Figure S4B). Healthy and EC groups presented a similar pattern of significantly more abundant taxa dominated by *Ruminococcaceae* and *Lachnospiraceae* members. Strikingly, ET and LT‐HR coincided in *Propionibacterium*, *Carnobacterium*, *Pseudomonas*, *Butyricicoccus*, *Dorea* and *Rothia* as exclusive abundant genera. By contrast, LT‐LR showed a different pattern to the rest of groups, with greater abundance of *Enterobacteriaceae* pathobionts as *Escherichia* (Figures S4A,B). Interestingly, when ET or LT‐HR were compared with LT‐LR, both first concurred in most of their abundant OTUs belonging to *Propionibacterium*, *Carnobacterium*, *Pseudomonas* and *Dorea*, but also *Blautia*, *Clostridium* and *Veillonella* genera. In addition, comparison between ET and LT‐HR showed the lowest number of differentially abundant OTUs (Figure 3B). All these results could be corroborated by an alternative method to calculate the effect size, LEfSe, obtaining the same characteristic abundant OTUs for each group (Figure 3C). Taxonomic relationships of phylogeny among all the OTUs obtained were displayed using a cladogram (Figure 3D). No significant additional information was obtained when abundance analyses were performed separating ileum and caecum samples (data not shown).

FIGURE 3Comparison of relative abundances of different microbiome taxa derived from operational taxonomic units (OTUs) obtained in gut mucosal samples of study subjects. DESeq2 and LefSe methods were used in order to detect differentially abundant OTUs. For DESeq2, one pseudocount was added to the raw counts before performing the analysis and a false discovery rate (FDR) threshold of .05 was considered. LefSe analysis was done with default parameters, using rarefied counts. (A) Diagram of accumulated bars showing OTUs from gut mucosal biopsies grouped in different phyla. The length of each coloured bar within the study groups represents the relative abundance (out of 1) of a bacterial phylum (according to the legend on the right). a: phylum with a significantly different relative abundance (*p* < .05) respect to the rest of the groups; b, c and d: phylum with a significantly different relative abundance (*p* < .05) respect to EC group. (B) Comparison of OTUs with significantly higher relative abundances obtained from gut‐mucosal microbiota of the three treated human immunodeficiency virus (HIV)‐groups when these groups were faced by pairs. These comparisons were selected among the total 10 comparisons established among the five study groups that can be seen at Figure S4A. Asterisks point to those OTUs that depict similarity between ET and LT‐HR groups (coinciding in these two groups when compared with LT‐LR), and dissimilarity among these two and LT‐LR. (C) LEfSe analysis result showing linear discriminant analysis (LDA) scores for the most representative differentially abundant OTUs (with a relative abundance more than three times higher in a group) found when gut samples of the five study groups were compared among them. (D) Cladogram depicting the phylogenetic relationships established between the different taxonomic levels corresponding to OTUs obtained by LEfSe analysis of the gut samples from study groups (coloured boxes on the right represent microbial families and genera, but not OTUs, to improve visualization). Kruskal–Wallis rank sum tests, followed by post hoc Tukey's procedure, were used to test for inter‐group differences in relative abundance. EC, elite controllers; ET, early‐treated; LT‐HR, late‐treated high recovery; LT‐LR, late‐treated low recovery; f, family; g, genus

**Figure:**
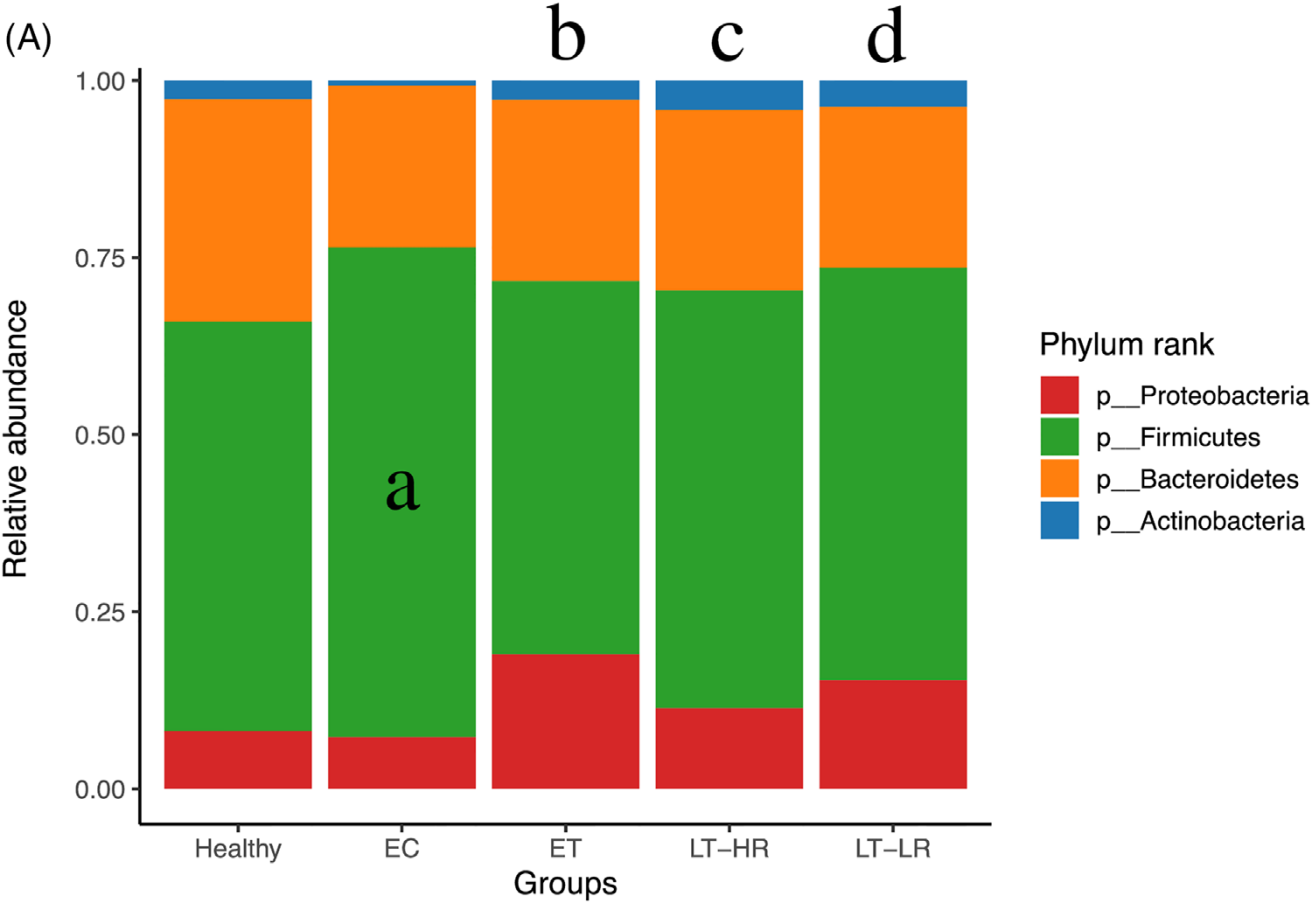

**Figure:**
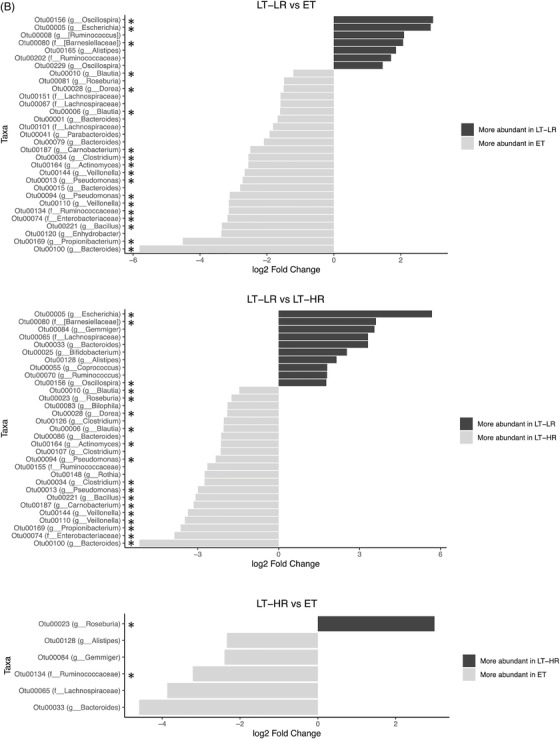

**Figure:**
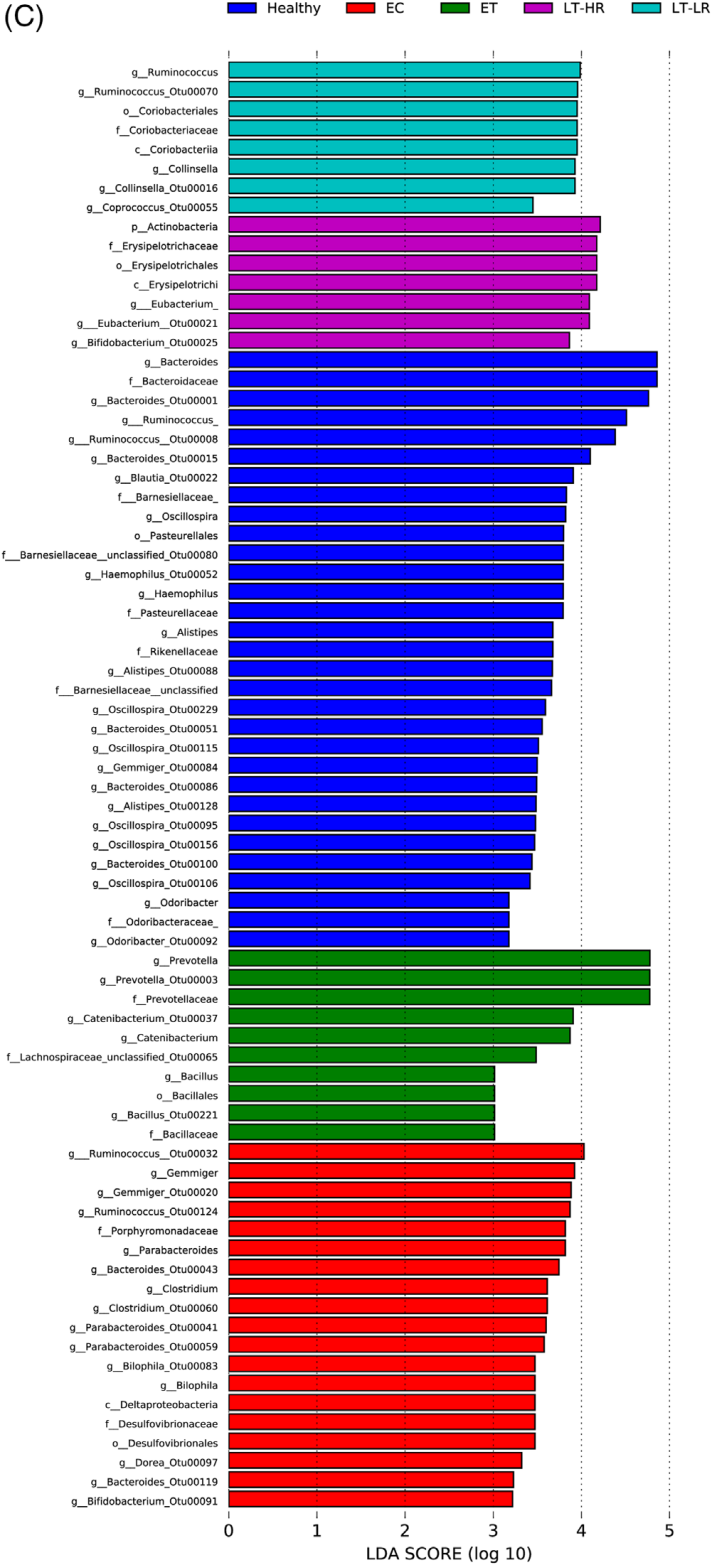

**Figure:**
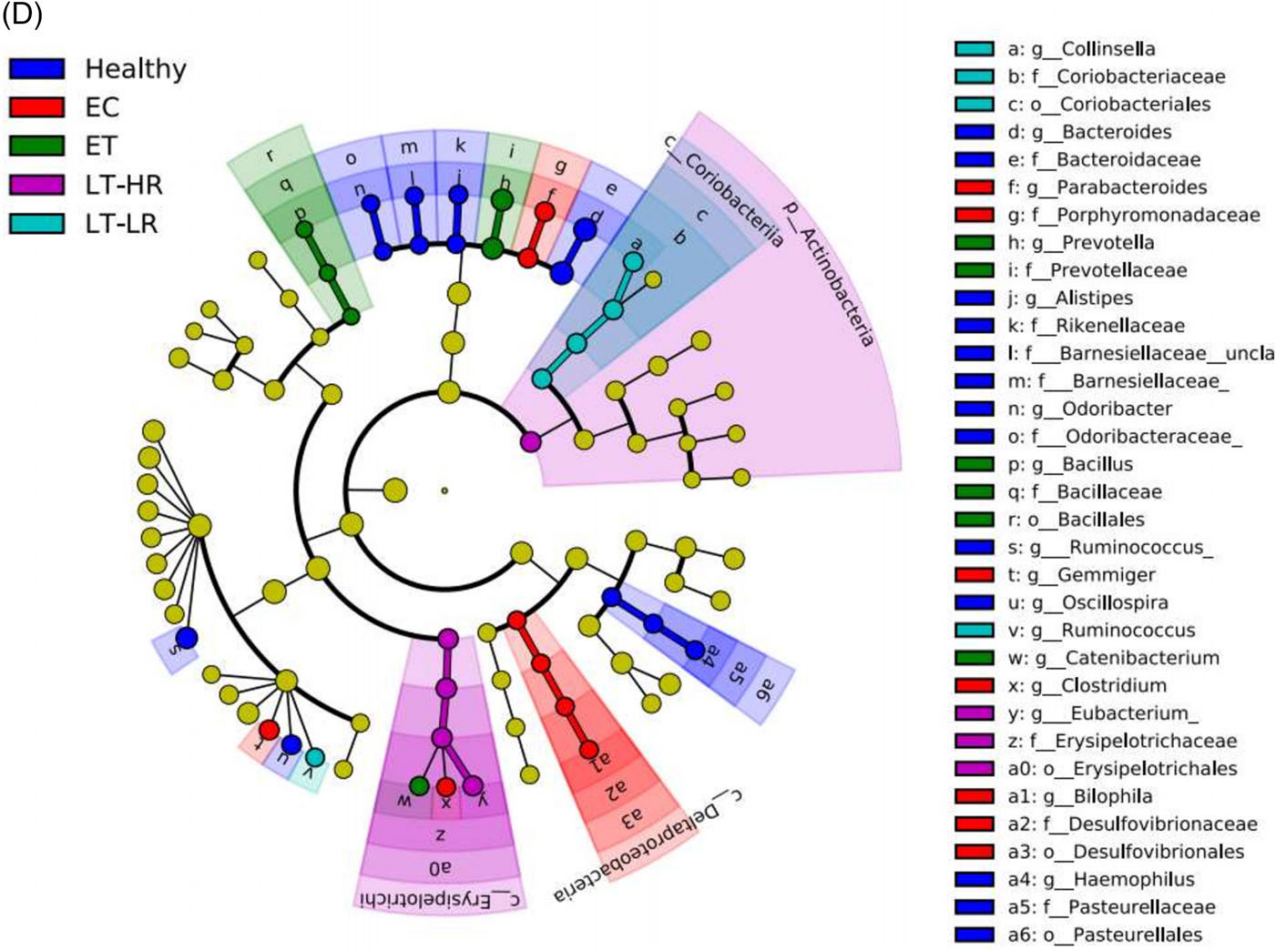

A random forest analysis was performed to find out OTUs whose relative abundance could classify treated‐subjects based on their immune status, regardless the gut location analysed. Again, ET and LT‐HR samples showed a large overlap when the three treated‐groups were analysed separately, so were grouped for further analysis. We chose the 14 most relevant OTUs (mean decrease accuracy, MDA > 5) (Figure 4A) for a multivariable logistic regression model that yielded a nine OTUs’ signature as the best to predict samples belonging to ET/LT‐HR or LT‐LR groups (Figure 4B). Using an receiver operating characteristic (ROC) curve, predictions of this OTUs‐based model gave an area under curve of .97.

**FIGURE 4:**
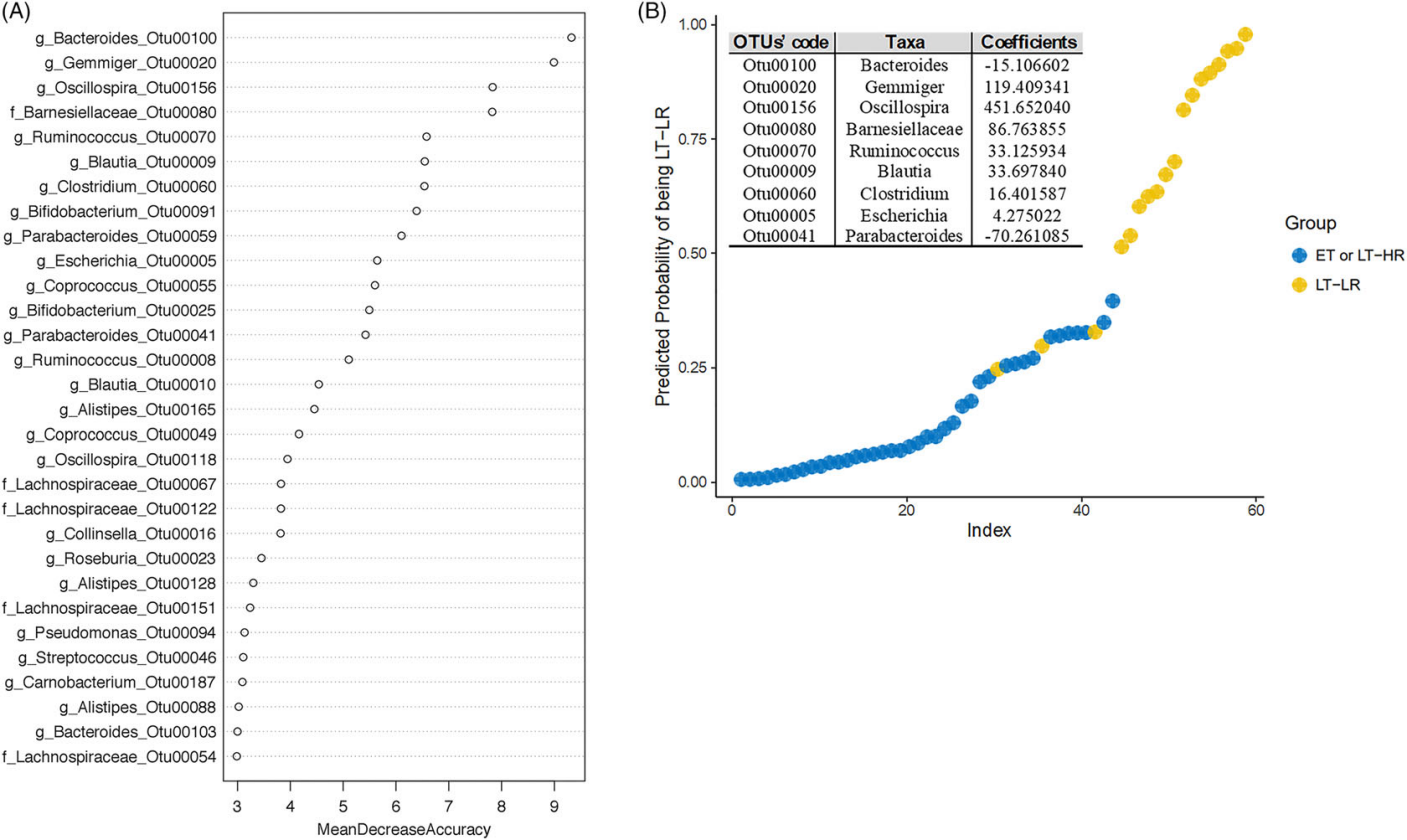
Calculations for an abundance‐based operational taxonomic units (OTUs) signature to predict immune progression of human immunodeficiency virus (HIV)‐infected subjects from their gut mucosal microbiota. Relative abundances from rarefied counts were used as input to 1000 random forests, and out‐of‐bag error was computed using the randomForest package in R. Initial analysis was performed with samples of the three treated HIV‐groups separated, but a classification error of 73% with LT‐HR samples, as many of them were assimilated as ET, and an out‐of‐bag error of 35% uncovered the great overlap existing between ET and LT‐HR groups. Once grouped together for further analyses, the out‐of‐bag error decreased to 12%. Given the multi‐collinearity of some OTUs, Lasso (L1‐norm) regularization was performed using R glmnet package, selecting the lambda through k‐3 cross‐validation, which minimizes the error in order to remove less relevant and multi‐collinear features (OTUs) before performing the logistic regression analysis. ROC curves were computed using InformationValue package in R. (A) Random forest (RF) analysis showing the best 30 OTUs to classify samples in either ET/LT‐HR or LT‐LR groups, showing their respective mean decrease accuracies (MDA). (B) Multivariable logistic regression model built from the 14 best predictors in RF (MDA > 5) yielding a signature of nine OTUs, whose log‐odds coefficients are shown in the table, that discriminate samples belonging to ET/LT‐HR or LT‐LR groups with a minimum misclassification error of 5% and area under curve (AUC) of .97 in an ROC analysis. ET, early‐treated; LT‐HR, late‐treated high recovery; LT‐LR, late‐treated low recovery; f, family; g, genus

Some peripheral (Figure S5, panel A) and gut mucosa‐related (Figure S5, panel B) parameters were compared in available samples of subgroups of subjects belonging to the different treated‐groups. Most of the inflammation markers, either soluble or tissue‐scored (Tables S1 and S2), as well as microbial translocation, immune activation and mucosal barrier damage parameters were higher in late‐treated groups, especially in non‐recoverers (LT‐LR).

A limitation of this study was the low number of samples, although being comparable with previous studies using gut‐mucosal samples in groups of HIV‐subjects. Such limitation made not possible to match groups by age and/or gender. Additionally, it would have been relevant to record data about transmission risk (i.e., men who have sex with men) and the additional study on metabolic pathways would have helped elucidate differential microbiome functions possibly associated to particular HIV‐groups.

In conclusion, this is the first report to analyse the potential association of the clinical phenotype of HIV‐subjects, regarding their CD4 status at cART onset and afterwards, with alterations in the microbiome composition. Also importantly, our study was performed in two different gut‐mucosal locations when most related studies are based on faecal samples.^4^, ^5^ Late‐treated‐subjects recovering CD4 under cART (LT‐HR) showed a partial restoration of the gut‐mucosal dysbiosis produced by HIV‐infection, being their microbiome composition similar to that of the more immunopreserved early‐treated‐subjects (ET). Such composition included *Propionibacterium*, *Carnobacterium*, *Pseudomonas* or *Dorea*, among others, as coinciding and exclusive abundant genera. In contrast, non‐recoverers (LT‐LR) appeared enriched in *Escherichia*, despite displaying all late‐treated‐subjects similar alpha‐diversity values in their microbiota. In addition, a nine OTUs‐based signature could be established for the non‐recovery clinical phenotype, also affected by more inflammation, immune activation and gut tissue damage. Early treatment and optimal cART regimen election^9^ seem to impact both, the composition of the gut‐mucosal microbiome and the clinical evolution of HIV‐subjects. Our descriptive study does not allow concluding about causality, and further approaches are encouraged to determine if modifying the gut‐microbiota composition could help the CD4 recovery. In that case, potential clinical interventions, as faecal microbiota transplant,^10^ are showing promising results at modifying gut microbiota structure to correct HIV‐associated dysbiosis (extended discussion at SI‐text).

## CONFLICT OF INTEREST

The authors declare no conflict of interests.

## Supporting information

Supporting information.
