# Supplementary material for: Partial restoration of gut‐mucosal dysbiosis in late‐treated HIV‐infected subjects with CD4 T‐cell recovery
Source: Clin Transl Med. 2022 Apr 5;12(4):e788. doi: 10.1002/ctm2.788 (PMC8982320; doi:10.1002/ctm2.788)

Supporting information

Partial restoration of gut-mucosal dysbiosis in late-treated HIV-infected subjects with CD4 T-cell recovery.

Letter-to-Editor with previous submission number CTM2-2021-11-2310

Running title: Gut-mucosal dysbiosis and CD4 recovery in HIV

Authors: Israel Olivas-Martínez[1], Isaac Rosado-Sánchez[1], Juan Antonio Cordero-Varela[2], Salvador Sobrino[3], Miguel Genebat[1], Inés Herrero-Fernández[1], Rocío Martínez de Pablos[4], Ana Eloísa Carvajal[4], Rocío Ruiz[4], Ana Isabel Álvarez-Ríos[5], María Fontillón-Alberdi[6], Ángel Bulnes-Ramos[1], Vanesa Garrido-Rodríguez[1], María del Mar Pozo-Balado[1], Manuel Leal[,7], Yolanda María Pacheco[1]

Affiliations:

1. Immunology Lab, Institute of Biomedicine of Seville, University Hospital Virgen del Rocío/CSIC/University of Seville, Seville, Spain

2. Bioinformatics and Computational Biology Service, Institute of Biomedicine of Seville, University Hospital Virgen del Rocío/CSIC/University of Seville, Seville, Spain

3. Digestive Service, University Hospital Virgen del Rocío, Seville, Spain

4. Biochemistry and Molecular Biology Department, Pharmacy, University of Seville, Spain; Institute of Biomedicine of Seville, University Hospital Virgen del Rocío/CSIC/University of Seville, Seville, Spain

5. Biochemistry Service, University Hospital Virgen del Rocío, Seville, Spain

6. Service of Pathological Anatomy, University Hospital Virgen del Rocío, Seville, Spain

7. Internal Medicine Service, Viamed-Santa Ángela Hospital, Seville, Spain

Correspondence to:

Yolanda M. Pacheco (ypacheco-ibis@us.es). Immunology Lab, Institute of Biomedicine of Seville (IBiS), Clinic Unit of Clinical Laboratories, Virgen del Rocío University Hospital, Avda. Manuel Siurot, s/n, PC: 41013, Seville, Spain. Tlf: +34 955 923 120

BACKGROUND

The compositional shift of the normal gut microbiome, known as dysbiosis, is an alteration commonly found in HIV-infected subjects.[1, 2] Dysbiosis is usually associated with an increase in OTUs belonging to Proteobateria phylum to the detriment of Firmicutes and Bacteroidetes, the most abundant microbiome members in the normal scenario.[3, 4] Another typical finding in HIV-subjects' microbiota consists of the increase in Prevotella and the decrease in Bacteroides genus, but the presence of this alteration is thought to be confounded by different sexual behaviours.[5, 6] In these studies, several luminal bacterial taxa have been reported to be linked to certain groups of HIV-infected subjects.[7, 8] Likewise, some inflammation and immune activation markers have been related to changes in gut microbiota of HIV-infected subjects,[9] with very scarce mentions so far to parameters evaluating the histological damage of the intestinal tissue in these subjects.[10]

To date, there is no consensus on a single sample type or gut location suitable for studying dysbiosis in HIV-subjects. Thus, sequencing and analysis of microbial 16S rRNA variable regions have been mainly performed in fecal material, likely due to its easier obtainment, rectal swabs and less frequently in mucosal biopsy samples of different gut locations.[11] Consequently, most of the microbiome studies rely on findings from fecal samples; however, when fecal and biopsy samples have been simultaneously studied, more and stronger changes were noted in the microbiota of gut-mucosal biopsies.[12, 13] Moreover, it is known that interactions with gut-associated lymphoid tissues (GALT) depend mostly on mucosal-specific rather than luminal/fecal microorganisms.[14, 15] In works using gut biopsies, colon has been the preferentially studied region,[16-18] but barely in comparison with other intestinal sites. In those last studies, only slight minor

differences have been reported between colon and ileum regarding microbiota composition, but not in relation to some site-restricted immune alterations found.[13, 19, 20]

These alterations associated with gut dysbiosis especially affect to late-diagnosed and/or late-treated individuals, representing 40-60% of HIV-subjects.[21] Among them, one-fourth of cases do not properly recover CD4 T-cell levels after combined antiretroviral therapy (cART) and suffer from more morbimortality.[22] Several studies have explored the effect of cART instauration on the gut microbiome of these immunodiscordant and other types of HIV-subjects,[23, 24] even checking individual drugs or combinations more specifically (reviewed in [25]). However, in treated-subjects, very few reports, based mainly on fecal samples, have addressed the association between immune recovery and gut dysbiosis with variable results.[5, 7, 26-30] Thus, the potential association between the immune status at cART onset and changes occurring as a result in the gut microbiome of HIV-infected subjects has not been explored yet.

Our hypothesis was that those HIV clinical phenotypes with poorer immune status (in terms of CD4 counts at cART onset and afterwards) would present a more dysbiotic microbiome in their gut mucosa. Therefore, our aim was to explore the potential specific dysbiosis in the gut-mucosal microbiome, comparing biopsies from two different locations (ileum and caecum), of HIV-infected subjects with different immunological profiles at the onset of cART and afterwards. Associations with parameters of inflammation, immune activation and gut tissue damage were also explored.

METHODS

Study subjects and samples

Biopsy samples of terminal ileum and caecum mucosa were taken from 38 HIV-infected and 10 healthy volunteers during routine colonoscopies performed at the Virgen del Rocío University Hospital between 2014 and 2017. Throughout the intervention, approximately ten-to-twelve pinch gut biopsies were obtained using cold disposable forceps (EndoJaw, FB-240U; Olympus Medical System Co, Tokio, Japan), as well as a peripheral blood sample collected the same day just before the medical procedure started. Exclusion criteria for this study were: having recent HIV rebounds, intestinal infections, cancer, active hepatitis C virus (HCV) infection or inflammatory processes. All the subjects enrolled in this study were properly informed and signed the corresponding informed consents either for the colonoscopy and the experimental study. The Comité de Ética de la Investigación de los Hospitales Universitarios Virgen Macarena-Virgen del Rocío approved this study, that was carried out following the European Union guidelines and the Declaration of Helsinki.

HIV-infected patients, all virologically suppressed (<50 HIV RNA copies/mL) at the date of colonoscopy, were classified into three groups depending on their immunological response upon receiving antiretroviral therapy as follows: early-treated (ET, n=14), who started cART with >250 CD4 T cells/mm^3 and remained >250 CD4 T cells/mm^3 two years later; late-treated high recovery (LT-HR, n=9), starting last cART with <250 CD4 T cells/mm^3 but reaching >250 CD4 T cells/mm^3 two years later; late-treated low-recovery (LT-LR, n=12), starting cART with <250 CD4 T cells/mm^3 and remaining <250 CD4 T cells/mm^3 two years later. Additionally, a reduced group of elite controllers (n=3), HIV-subjects with spontaneous virological suppression in the absence of cART, was also included. Control healthy subjects (n=10) were individuals with

similar age and sex characteristics to those of treated HIV-subjects, without infectious or inflammatory pathologies (Fig. 1). Participants were recruited trying to have homogeneous groups as possible attending age and sex, however it has to be noted that these clinical phenotypes normally have intrinsic differences regarding these criteria. On the other hand, since colonoscopy is an invasive medical procedure, the number of subjects recruited was importantly limited to those volunteers who freely agreed to participate.

Measurement of blood parameters

Absolute counts of CD4 and CD8 T-cells, along with other blood cells, were determined in fresh samples with an Epics XL-MCL flow cytometer (Beckman Coulter, California, USA). Quantitative polymerase chain reaction (qPCR) was used to determine plasma HIV RNA levels (PCR; COBAS Ampliprep/COBAS Taqman HIV test, Roche Molecular Systems, Basel, Switzerland) according to manufacturer's instructions and with a detection limit of 20 HIV RNA copies/mL. Plasma samples were also used to measure levels of Lipopolysaccharide binding protein (LBP) by enzyme-linked immunosorbent assay (Human ELISA kit, Hycult Biotech, Uden, The Netherlands). Highly sensitive C-reactive protein (hsCRP) and ß2-microglobulin were assessed with an immunoturbidimetric serum assay, whereas other serum parameters were determined by colorimetric assay, all using a Cobas 701 (Roche Diagnostics, Mannheim, Germany).

Immunophenotyping

Peripheral blood mononuclear cells (PBMCs) were fixed for staining of different surface markers with the following antibodies: anti-CD3, anti-CD4, anti-CD8, anti-CD38 (BD Biosciences, USA) and anti-HLA-DR (Biolegend, USA). Activated T cells were defined as HLA-DR$^+$CD38$^+$CD4$^+$ or HLA-DR$^+$CD38$^+$CD8$^+$. LIVE/DEAD fixable Aqua Blue Dead Cell Stain (Life Technologies, USA) was used to determine living cells. A

minimum of 100,000 events of total lymphocytes were acquired. Flow cytometry was performed on an LSR Fortessa (BD Biosciences, USA) and analysed using FlowJo version 9.3 (TreeStar). Data were expressed as frequencies (%) of cells expressing the different studied markers.

Damage on mucosal integrity

Gut-mucosal tissues embedded in paraffin were sectioned (5 μm) and then stained with hematoxylin-eosin. To assess histological damage in ileum and caecum biopsies, a semi-quantitative scale (0-3 scores) was used taking into account different typical structures present in the intestinal mucosa of each anatomical location and following established score guidelines for complete intestinal sections of human and mouse[*] biopsies. Histological evaluation was carried out by a trained pathologist who was blinded to clinical data of the study subjects using a light microscope (Olympus BX61, Japan). Some of the parameters scored with this scale were: destruction of epithelium and glands, loss of Goblet cells, villi destruction, glandular crypts expanded, depletion of Paneth cells or inflammatory cells infiltration (Tables S1 and S2). Higher scores in this scale are indicative of higher damage on mucosal integrity. The total damage score for ileum and caecum of each subject (minimum is 0 and maximum 15) is the sum of the subscores obtained from the different histological parameters evaluated. The gut histological scores are represented as median [IQR] values.

[*] Erben, U. et al. A guide to histomorphological evaluation of intestinal inflammation in mouse models. *Int. J. Clin. Exp. Pathol.* **7**, 4557-4576 (2014).

	Parameters for Caecum	Epithelial destruction	Crypt/Gland destruction	Crypt dilatation	Goblet loss or reduction	Mucosal infiltration
SCORE	0	Normal morphology	Normal morphology	Normal morphology	Normal appearance	Normal
	1	Local destruction	Local destruction	Slightly dilated / local zone	Slightly reduced	Slightly increased
	2	Zonal destruction	Zonal destruction	Moderate dilatation / several zones	Moderately reduced	Moderately increased
	3	Extensive destruction	Extensive destruction	Severe dilatation / most zones	Severely reduced or absent	Severely increased

Table S1. Criteria for a semi-quantitative histological assessment of the loss of mucosal integrity in caecum.

	Parameters for T. Ileum	Intestinal villi	Goblet cells	Crypt dilatation	Paneth cells	Mucosal infiltration
SCORE	0	*Glove finger*	Normal appearance	Normal morphology	Normal appearance	Normal
	1	Slightly widened and shortened	Slightly reduced	Slightly dilated / local zone	Slightly reduced	Slightly increased
	2	Moderately widened and shortened	Moderately reduced	Moderate dilatation / several zones	Moderately reduced	Moderately increased
	3	Severely widened and shortened	Severely reduced or absent	Severe dilatation / most zones	Severely reduced or absent	Severely increased

Table S2. Criteria for a semi-quantitative histological assessment of the loss of mucosal integrity in terminal ileum.

Immunofluorescence in gut tissues

Expression of Caspase-3, and intestinal barrier proteins as Zonulin-1 (ZO-1) and Mucin (Muc2) was assessed by indirect immunofluorescence. Briefly, paraffin-embedded sections of gut tissues (5 µm) were cut using a cryostat at -20 ºC and mounted on gelatin-coated slides. After permeabilization with 1% Triton X-100 in PBS for 1 h, sections were blocked in 5% (w/v) BSA, 1% Triton X-100 in PBS for another 1 h, and then incubated overnight at 4 °C with the primary antibody diluted in 1% (w/v) BSA, 1% Triton X-100 in PBS. After that, slides were rinsed for 1 h in PBS with 0.1% Triton X-100, incubated 1 h with the corresponding secondary antibody diluted in 1% (w/v) BSA, 0.1% Triton X-100 in PBS, and then rinsed again in 0.1% Triton X-100 in PBS for 1h.

Images were acquired using an inverted ZEISS LSM 7 DUO confocal laser scanning microscope with the same laser intensity and gain conditions. Immunofluorescence intensity was quantified using Image-J free software package (http://rsb.info.nih.gov/ij/download.html) and calculated as a percentage of area relative to Hoechst fluorescence. All images were taken with the same Z, so that they had the same thickness in the microscope.

Microbiome analyses

DNA of gut tissue samples (stored at liquid nitrogen) was extracted from ileum and caecum biopsies using QIAamp PowerFecal Pro DNA Kit (QIAGEN, Hilden, Germany) by an automated procedure (QIACUBE, QIAGEN, Hilden, Germany). DNA quantification and extraction quality were evaluated using a Nanodrop 2000 C (Thermo Fisher Scientific, Waltham, Massachusetts, USA). This ileum and caecum mucosal DNA was subsequently used as a template to perform microbiome analysis. Eleven mucosal biopsy samples were discarded before sequencing due to poor DNA quality at the extraction process. The remaining 85 ileum and caecum DNA samples were used to

perform microbiome 16S rRNA sequencing through MiSeq System technology (Illumina, San Diego, CA, USA) using v3 reagents (600 cycles) and 20% PhiX as internal control. Primers used for library preparation are specific of V3 and V4 variable regions of the prokaryotic 16S ribosomal RNA gene, as was described by Klindworth *et al.*[*]

Sequencing data were processed with Mothur (version 1.43.0), using SILVA (non-redundant version 138) and Greengenes (version 13_8_99) for aligning and taxonomic purposes, respectively. OTU picking was performed at 97% sequence similarity to detect subgenera.

Subsequent data analysis was accomplished using the *Phyloseq* package (R version 3.6.3). Only bacterial taxa present at least in 50% of the samples analysed were considered. Several alpha and beta diversity measures were calculated using *Phyloseq* and *Vegan* packages. Non-metric multidimensional scaling (NMDS) was used to represent the difference in sample composition among groups, choosing Morisita-Horn index as beta diversity index since it yielded the lowest stress value.

In order to detect differentially abundant taxa, LefSe and DESeq2 methods were used. LefSe analysis was done with default parameters, using rarefied counts. For DESeq2, one pseudocount was added to the raw counts before performing the analysis and a False Discovery Rate (FDR) threshold of 0.05 was considered to select differentially abundant OTUs between clinical groups.

Alternatively, a random forest analysis was performed to discover a subset of OTUs which accurately classified samples into either ET/LT-HR or LT-LR group, via *randomForest* package in R. Relative abundances from rarefied counts were used as input to 1000 random forests and Out-of-Bag error was computed. Then, a multivariable logistic regression model was built to obtain a signature allowing for a good predictor of belonging to one group or another, depending on the relative abundances of the OTUs

with a mean decreased accuracy higher than 5 in the random forest. For this purpose, given the multi-collinearity of some OTUs, Lasso (L1-norm) regularization was performed using R *glmnet* package, selecting the *lambda* through k-3 cross-validation which minimizes the error, to remove less relevant and multi-collinear features (OTUs). ROC curves were computed using *InformationValue* package in R.

R code used to do data analysis of the microbiota present in ileum and caecum mucosal biopsy samples from different study groups is detailed in Annex S1.

[*] Klindworth, A. et al. Evaluation of general 16S ribosomal RNA gene PCR primers for classical and next-generation sequencing-based diversity studies. *Nucleic Acids Res.* **41**, e1 (2013).

Statistics

Continuous variables are expressed as medians and interquartile ranges (IQR), whereas categorical ones are expressed as number and percentages (%). Shapiro-Wilk test was used to evaluate normality of data analysed. Kruskal-Wallis and post-hoc Dunn's tests were used to estimate differences in blood and tissue parameters among different groups. Chi-square (χ^2) or Fischer's exact test were applied to search for significant differences among categorical clinical data. Correlation between diversity values and different gut locations was assessed using Spearman's Rho Coefficient. Kruskal-Wallis rank sum tests, followed by post-hoc Tukey's procedure, were used to test for differences in clinical variables, alpha-diversity measures and non-metric multidimensional scaling (NMDS) axes among study groups. Differences in beta-diversity were tested using PERMANOVA and HOMOVA procedures. A p-value <0.05 was considered statistically significant. Prism, version 7.0 (GraphPad Software, Inc.) and R statistical software (version 3.6.3) were both used for statistical analysis and generation of the graphs.

RESULTS

Baseline characteristics of the study cohort

The main clinical features of the patients are detailed in Table S3. Although the medium age of patients was 50 years-old, a significant difference was found probably due to the younger age of ET patients; however, it disappeared in pairwise comparisons or after grouping all HIV-groups against healthy individuals (Annex S2). Study groups, mainly composed of men, with the exception of healthy group, also showed differences in time from diagnosis, as well as in the presence of past HCV infections, which were both greater in LT-LR and EC patients. Moreover, time under cART was significantly lower in early-treated. Regarding the type of cART, nucleoside reverse transcriptase inhibitors (NRTI) were the preferred treatment choice although accompanied by protease inhibitors (PI) in case of ET and LT-HR groups, or by integrase strand transfer inhibitors (INSTI) in case of LT-LR patients; however, there were no significant differences. As expected, CD4 counts and CD4/CD8 ratio were different among groups, either just before their last cART or at the colonoscopy date. In both parameters, LT-LR patients group always showed the lowest levels.

	Healthy (n=10)	EC (n=3)	ET (n=14)	LT-HR (n=9)	LT-LR (n=12)	P-value
**Age at biopsy (years)**	57 (46-62)	52 (49–55)	42 (32–52)	49 (45-66)	54 (51-56)	***0.037***
**Male sex, n (%)**	4 (40%)	2 (67%)	14 (100%)	9 (100%)	9 (75%)	***0.003***
**Time since diagnosis (years)**	-	31 (22-32)	6 (4-11)	9 (6-21)	16 (7-25)	***0.009***
**AIDS event (%)**	-	1 (33%)	2 (14%)	4 (44%)	7 (58%)	0.128
**Past HCV infection (%)**	-	3 (100%)	1 (7%)	1 (11%)	5 (42%)	***0.004***
**Total time on cART (months)**	-	279 (211-348)	62 (39-130)	103 (75-201)	171 (60-259)	***0.016***
**cART regimen at biopsy (drug types)**						0.197
NRTI	-	-	7 (50%)	5 (56%)	11 (92%)	
NNRTI	-	-	2 (14%)	2 (22%)	2 (17%)	
INSTI	-	-	7 (50%)	2 (22%)	8 (67%)	
PI	-	-	7 (50%)	5 (56%)	0 (0%)	
CCR5 antagonist	-	-	5 (36%)	4 (44%)	3 (25%)	

**Time since cART onset (months)**	-	-	59 (37-130)	102 (73-163)	68 (56-159)	0.184
**CD4 count before cART (cells/mm^3)**	-	-	441 (377-553)	46 (26-138)	20 (10-91)	**< *0.0001***
**CD4/CD8 ratio before cART (value)**	-	-	0.46 (0.3-0.62)	0.17 (0.03-0.51)	0.05 (0.03-0.11)	**< *0.0001***
**CD4 count at biopsy (cells/mm^3)**	832 (734-858)	758 (346-806)	955 (770-1181)	590 (387-772)	251 (180-312)	**< *0.0001***
**CD4/CD8 ratio at biopsy (value)**	2.16 (1.58-3.45)	0.86 (0.65-1.2)	1.33 (0.99-1.69)	0.92 (0.52-1.28)	0.44 (0.23-0.65)	**< *0.0001***

Table S3. Characterization of subjects from the study groups.

Quantitative variables are expressed as median [IQR] and categorical variables are expressed as the number of cases (%). Kruskall Wallis test was applied and *p* values < 0,05 were considered statistically significant and are shown in bold. ET: early-treated; LT-HR: late-treated high recovery; LT-LR: late-treated low recovery; EC: elite controllers; AIDS: acquired immune deficiency syndrome; HCV: hepatitis C virus; cART: combined antiretroviral therapy; NRTI: nucleoside reverse transcriptase inhibitor; NNRTI: non-nucleoside reverse transcriptase inhibitor; INSTI: integrase strand transfer inhibitor; PI: protease inhibitor; CCR5: chemokine receptor C-C type 5. Chi-square (χ^2) or Fischer's exact test and Kruskal-Wallis were the statistical tests used to search for significance.

Similar microbiome diversity between different gut locations in mucosal biopsy samples of all study groups

Sequencing of the microbiome present in ileum and caecum samples (n=48, for each location; total=96) from patients of different study groups yielded an average of 64,741 sequences per sample. Samples with less than 7,000 sequences (n=11; corresponding to six treated-subjects) were excluded from the analysis because their alpha-diversity values were fairly lower compared with the rest. Once analysed, no significant differences appeared in the observed number (Sobs index) or richness (Chao1 and Ace indexes) of OTUs found among HIV (EC, ET, LT-HR and LT-LR) and healthy individuals, regardless the biopsied intestinal area (Fig. 2A) or patient sex (Fig. S1). The results were similar when alpha-diversity (intra-group OTUs' richness and evenness) was estimated through Shannon and Simpson indexes, not observing differences among the study groups both in ileum and caecum mucosal samples or comparing by sex (Fig. 2A and S1). Even so, healthy and EC, the groups with more percentage of women, showed higher mean values of richness and diversity than ET and LT HIV-groups. A strong positive association was found ($r>0.8; p<0.0001$) between ileum and caecum samples of all study groups in terms of alpha-diversity (Fig. 2B), which still remained when each group was independently analysed (data not shown).

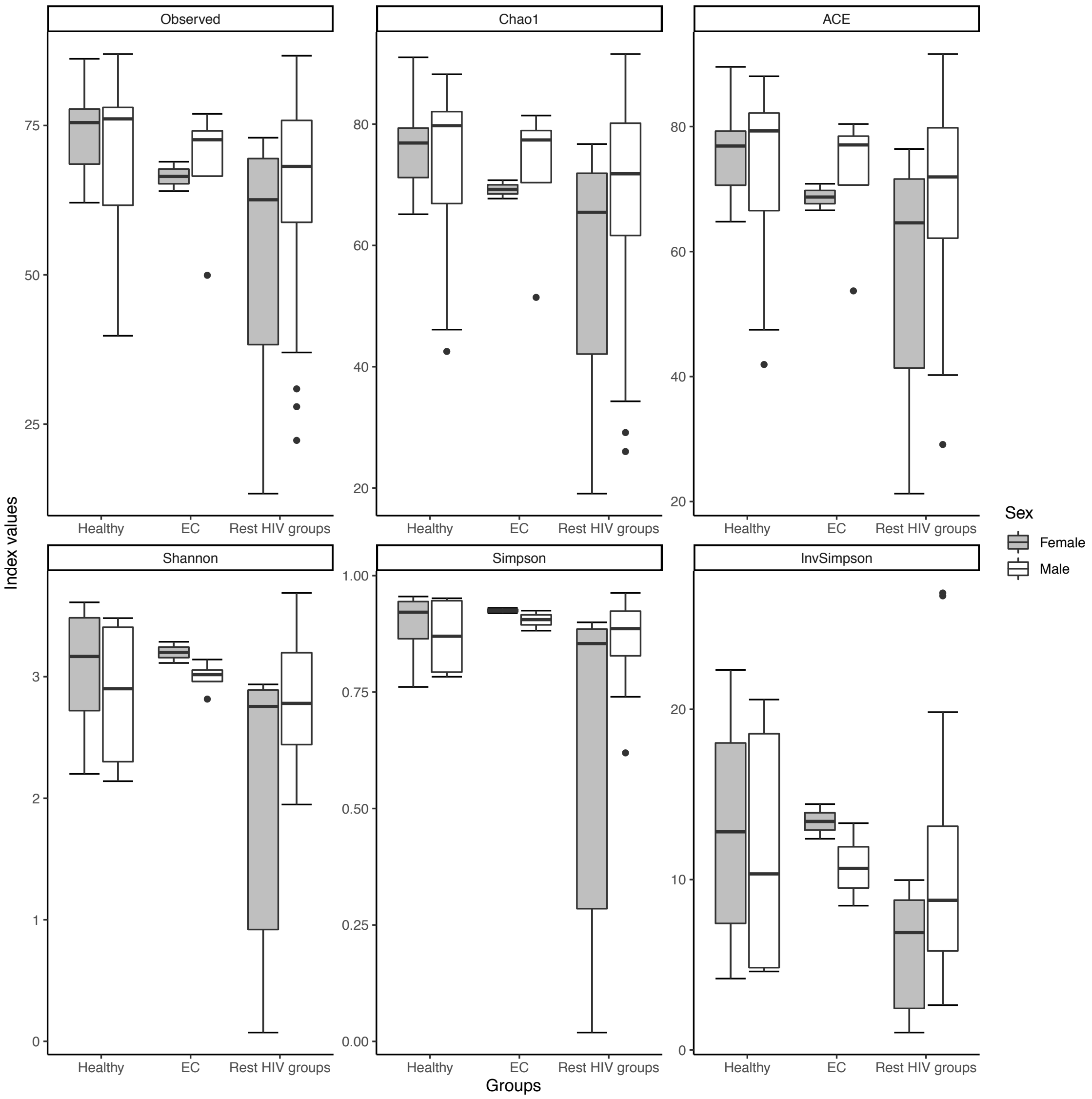

Figure S1. Differences in values of richness and alpha diversity indexes for gut microbiome samples from healthy, EC and rest of HIV (ET, LT-HR and LT-LR) groups compared by patients' sex. (dots represent outliers).

To estimate beta-diversity (inter-group OTUs' variation) of the gut microbiota, a NMDS analysis was employed, being ileum and caecum samples from healthy and EC groups clustered together in comparison to the rest HIV-groups (PERMANOVA, *p<0.001*; HOMOVA, *p<0.041*) (Fig. 2C). These results were confirmed calculating differences by axis, since healthy and EC groups showed significant distances with ET and LT HIV-groups, especially when NMDS1 axis was analysed (Fig. 2D). As for alpha

diversity, beta diversity did not show relevant differences when comparing ileum and caecum samples (Fig. S2). Thus, subsequent abundance analyses were performed without separating by gut location.

A

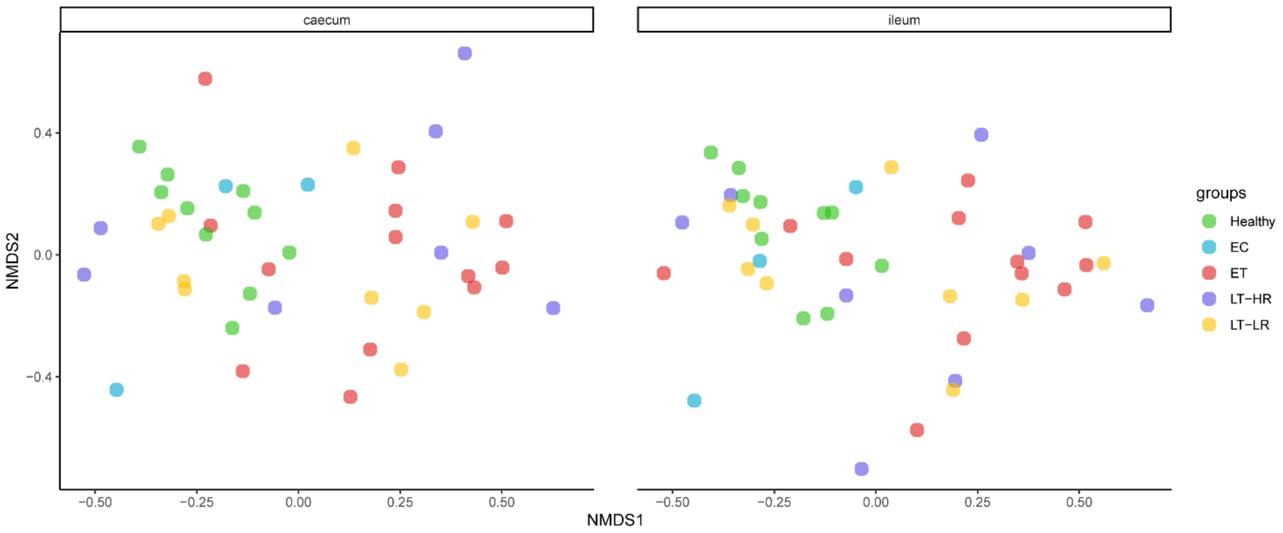

B

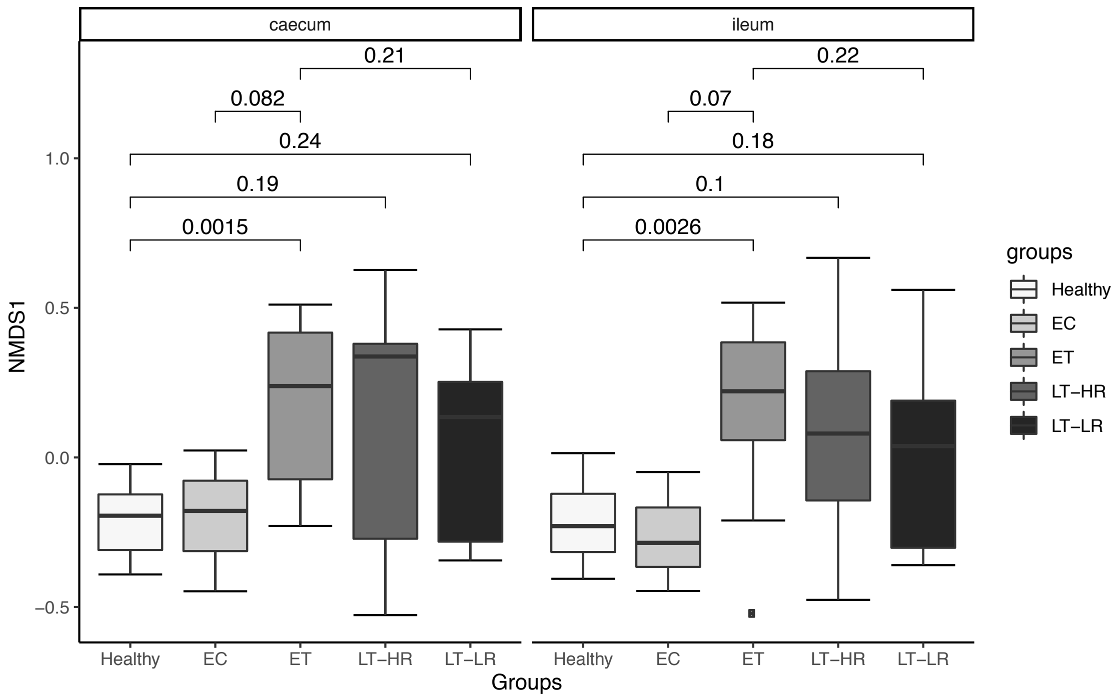

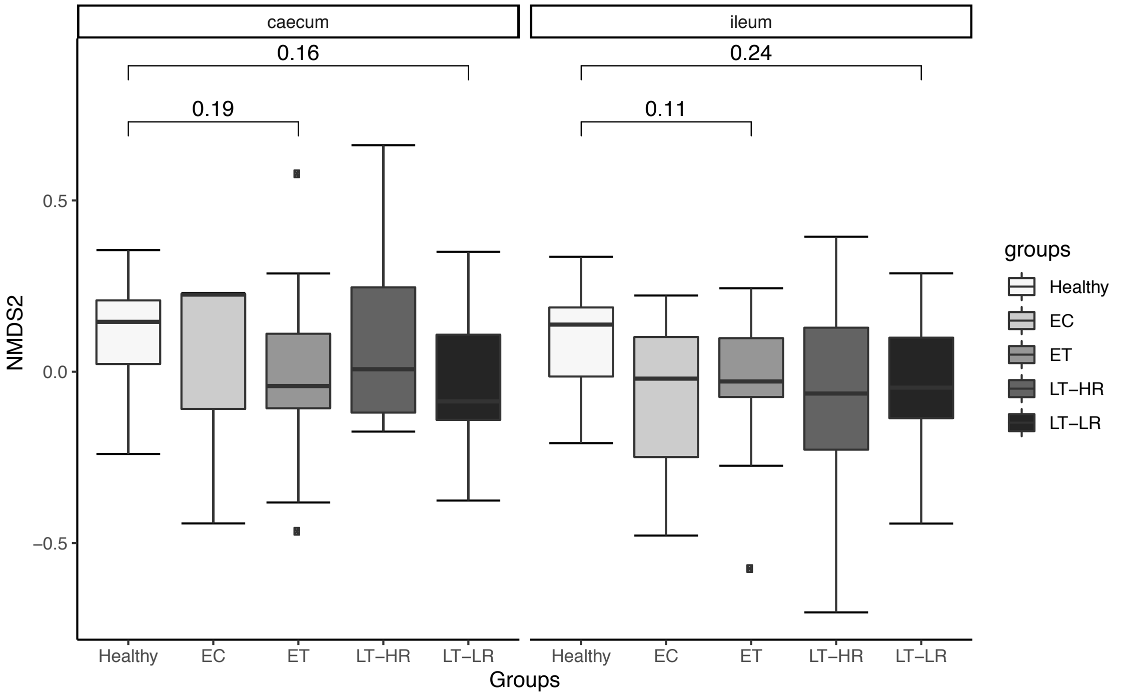

Figure S2. Non-metric multidimensional scaling (NMDS) analysis for beta diversity of the gut-mucosal microbiota from samples of the five study groups by anatomical location (ileum or caecum), either grouped (A) or separated by axes (NMDS1 and NMDS2) (B).

Gain of Proteobacteria at high taxonomic levels of the gut-mucosal microbiome from treated-subjects

Only thirty-six taxa presented a significantly different relative abundance in all comparisons done among the five study groups, although the corresponding OTUs were differentially distributed depending on the two groups being compared. At higher taxonomic levels, Firmicutes (with 53% of OTUs identified), Proteobacteria (19%), Bacteroidetes (17%) and Actinobacteria (11%) were the main phyla found in gut-mucosal samples isolated from all studied subjects' groups. Specifically, EC group showed the highest relative abundance of Firmicutes phylum in comparison with the rest of the groups ($p \leq 0.07$), but lower abundance of Actinobacteria compared to ET ($p=0.04$), LT-HR ($p=0.006$) and LT-LR ($p=0.01$) groups, respectively (Fig. 3A). At class level, more

abundant OTUs mainly belonged to Clostridia, Bacteroidia and Gammaproteobacteria (Fig. S3). When significantly different OTUs were analysed at this level, EC group presented the lowest abundance of Coriobacteriia (*p≤0.017*), which was the predominant class in LT-LR, but higher quantity of Deltaproteobacteria than ET (*p=0.017*) and LT-LR (*p=0.03*) groups. In addition, healthy group had more relative abundance of Bacteroidia than late-treated HIV-groups (*p=0.002 vs LT-HR* and *p=0.007 vs LT-LR*) and, by contrast, less presence of Bacilli and Erysipelotrichi than early and late-treated HIV-patients (*p≤0.03*). On the other hand, LT-HR group showed less Clostridia (*p=0.03*) but more Actinobacteria (*p=0.006*) than healthy group (Fig. S3).

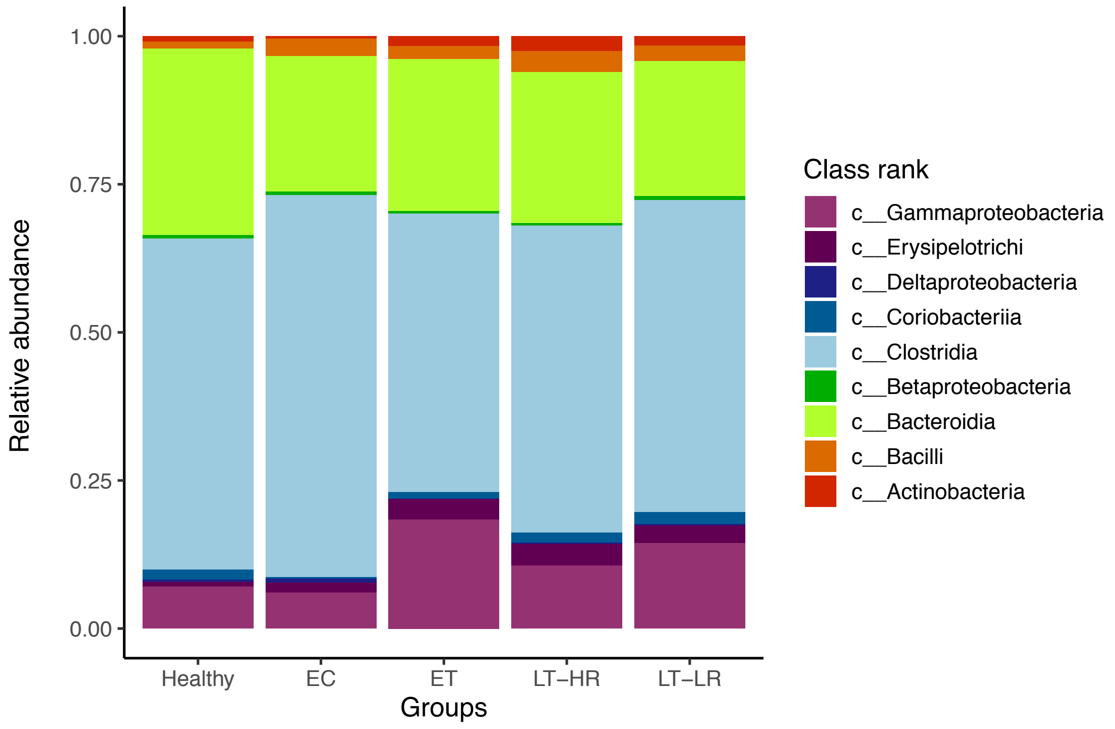

Figure S3. Diagram of accumulated bars showing operational taxonomic units' (OTUs) relative abundance for the gut mucosal biopsies analysed. The length of each coloured bar within the different study groups represents the relative abundance (out of 1) of a bacterial class (according to the legend on the right). Significant differences (p<0.05) found using Kruskal-Wallis rank sum tests, followed by post-hoc Tukey's procedure, are detailed in the manuscript above.

Presence of similar patterns of differentially abundant OTUs in the gut-mucosal microbiota from healthy/EC and ET/LT-HR patients after comparing study groups

Data about more specific taxonomic levels revealed that Lachnospiraceae, Ruminococcaceae, Enterobacteriaceae and Clostridiaceae were the most represented microbiota families to which OTUs belonged in abundance comparisons among study groups (Fig. S4A). We tried to summarize all data obtained in these ten comparisons by doing a taxonomic classification of groups of OTUs in their respective family and/or genus taxa (Fig. S4B). After that, we observed that Lachnospiraceae family and its members were less fold-times relatively abundant in late-treated groups (LT-HR and LT-LR) than in ET, EC and healthy groups. Relative abundance of Ruminococcaceae members followed a more homogeneous pattern as it resulted to be significantly higher in comparisons of each of the five study groups; whereas, Enterobacteriaceae was only more relatively abundant in ET, LT-HR and LT-LR HIV-groups when compared between them or with the other groups. By contrast, Clostridiaceae members (Clostridium and Butyricicoccus) were particularly less abundant in LT-LR. Finally, at genus level, Prevotella and Eubacterium were clearly more abundant in HIV-groups, except EC, while Bacteroides, even though being present in all study groups, was more differentially abundant in healthy subjects. Healthy and EC groups presented a similar pattern of significantly more abundant taxa in relation to the rest of the groups, both with Lachnospiraceae and Ruminococcaceae members, Clostridium, Bacteroides and Gemmiger, as well as Lachnospira, Ruminococcus and Roseburia, although these last three were predominantly abundant in healthy (Ruminococcus) or EC (Lachnospira and Roseburia), respectively. On the contrary, some microorganisms, like Streptococcus and Catenibacterium, were abundant in all the groups except healthy, and the same occurred with Haemophilus, among others, for EC group. ET and LT-HR shared almost all their most abundant taxa, with six bacterial genera, Propionibacterium, Carnobacterium,

Pseudomonas, Butyricicoccus, Dorea and Rothia been exclusively more abundant in comparisons of both groups, and not being present either in the rest of HIV (LT-LR and EC) or healthy subjects. Similarly, Bacillus, Blautia, Veillonella and Clostridium appeared as more abundant mainly in comparisons of both ET and LT-HR groups; however, these genera were also differentially abundant in comparisons of other study groups. On the other hand, LT-LR showed a different pattern to the rest of the groups, with greater abundance of Escherichia and more relative abundance of Oscillospira and Bifidobacterium in more comparisons than the other study groups; Barnesiellaceae and Coprococcus were also abundant in this group, although both taxa appeared in comparisons of ET and healthy groups, respectively (Figs. S4A and S4B). Interestingly, when ET or LT-HR were compared with LT-LR at OTUs level, both first concurred in most of their abundant OTUs belonging to Propionibacterium, Carnobacterium, Pseudomonas and Dorea, but also Blautia, Clostridium and Veillonella genera. In addition, comparison between ET and LT-HR showed the lowest number of differentially abundant OTUs (Fig.3B).

A

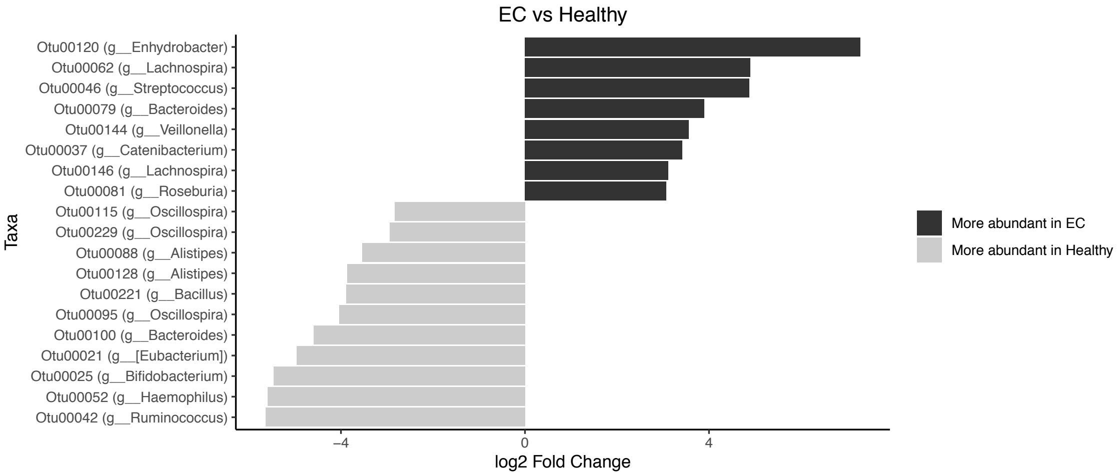

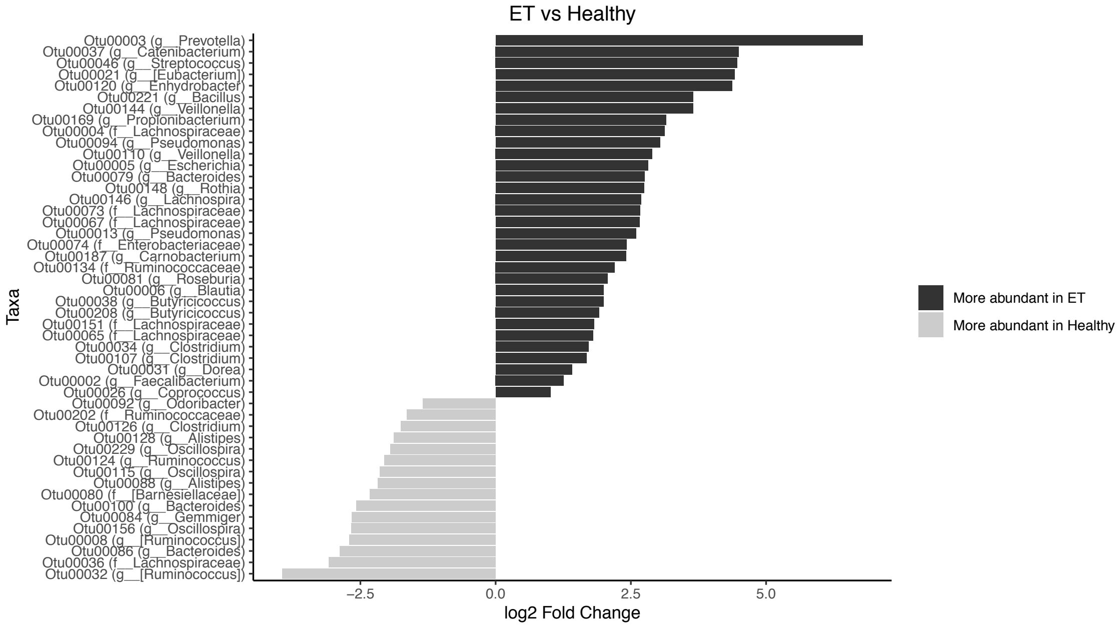
ET vs Healthy
Otu00003 (g__Prevotella)
Otu00037 (g__Catenibacterium)
Otu00046 (g__Streptococcus)
Otu00021 (g__[Eubacterium])
Otu00120 (g__Enhydrobacter)
Otu00221 (g__Bacillus)
Otu00144 (g__Veillonella)
Otu00169 (g__Propionibacterium)
Otu00004 (f__Lachnospiraceae)
Otu00094 (g__Pseudomonas)
Otu00110 (g__Veillonella)
Otu00005 (g__Escherichia)
Otu00079 (g__Bacteroides)
Otu00148 (g__Rothia)
Otu00146 (g__Lachnospira)
Otu00073 (f__Lachnospiraceae)
Otu00067 (f__Lachnospiraceae)
Otu00013 (g__Pseudomonas)
Otu00074 (f__Enterobacteriaceae)
Otu00187 (g__Carnobacterium)
Otu00134 (f__Ruminococcaceae)
Otu00081 (g__Roseburia)
Otu00006 (g__Blautia)
Otu00038 (g__Butyricicoccus)
Otu00208 (g__Butyricicoccus)
Otu00151 (f__Lachnospiraceae)
Otu00065 (f__Lachnospiraceae)
Otu00034 (g__Clostridium)
Otu00107 (g__Clostridium)
Otu00031 (g__Dorea)
Otu00002 (g__Faecalibacterium)
Otu00026 (g__Coprococcus)
Otu00092 (g__Odoribacter)
Otu00202 (f__Ruminococcaceae)
Otu00126 (g__Clostridium)
Otu00128 (g__Alistipes)
Otu00229 (g__Oscillospira)
Otu00124 (g__Ruminococcus)
Otu00115 (g__Oscillospira)
Otu00088 (g__Alistipes)
Otu00080 (f__[Barnesiellaceae])
Otu00100 (g__Bacteroides)
Otu00084 (g__Gemmiger)
Otu00156 (g__Oscillospira)
Otu00008 (g__[Ruminococcus])
Otu00086 (g__Bacteroides)
Otu00036 (f__Lachnospiraceae)
Otu00032 (g__[Ruminococcus])
Taxa
−2.5
0.0
2.5
5.0
log2 Fold Change
More abundant in ET
More abundant in Healthy

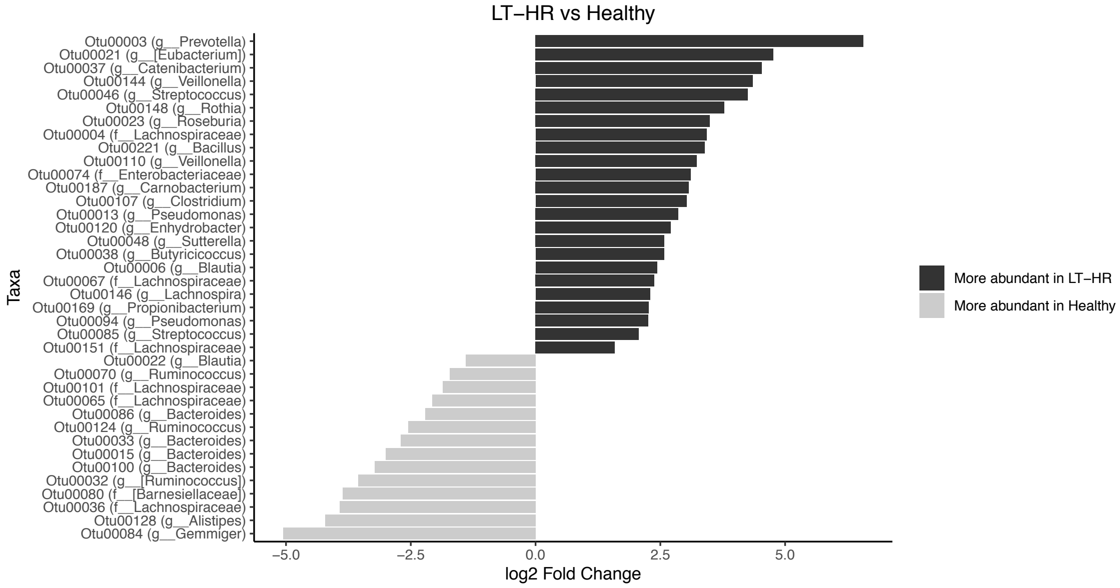
LT−HR vs Healthy
Otu00003 (g__Prevotella)
Otu00021 (g__[Eubacterium])
Otu00037 (g__Catenibacterium)
Otu00144 (g__Veillonella)
Otu00046 (g__Streptococcus)
Otu00148 (g__Rothia)
Otu00023 (g__Roseburia)
Otu00004 (f__Lachnospiraceae)
Otu00221 (g__Bacillus)
Otu00110 (g__Veillonella)
Otu00074 (f__Enterobacteriaceae)
Otu00187 (g__Carnobacterium)
Otu00107 (g__Clostridium)
Otu00013 (g__Pseudomonas)
Otu00120 (g__Enhydrobacter)
Otu00048 (g__Sutterella)
Otu00038 (g__Butyricicoccus)
Otu00006 (g__Blautia)
Otu00067 (f__Lachnospiraceae)
Otu00146 (g__Lachnospira)
Otu00169 (g__Propionibacterium)
Otu00094 (g__Pseudomonas)
Otu00085 (g__Streptococcus)
Otu00151 (f__Lachnospiraceae)
Otu00022 (g__Blautia)
Otu00070 (g__Ruminococcus)
Otu00101 (f__Lachnospiraceae)
Otu00065 (f__Lachnospiraceae)
Otu00086 (g__Bacteroides)
Otu00124 (g__Ruminococcus)
Otu00033 (g__Bacteroides)
Otu00015 (g__Bacteroides)
Otu00100 (g__Bacteroides)
Otu00032 (g__[Ruminococcus])
Otu00080 (f__[Barnesiellaceae])
Otu00036 (f__Lachnospiraceae)
Otu00128 (g__Alistipes)
Otu00084 (g__Gemmiger)
Taxa
−5.0
−2.5
0.0
2.5
5.0
log2 Fold Change
More abundant in LT−HR
More abundant in Healthy

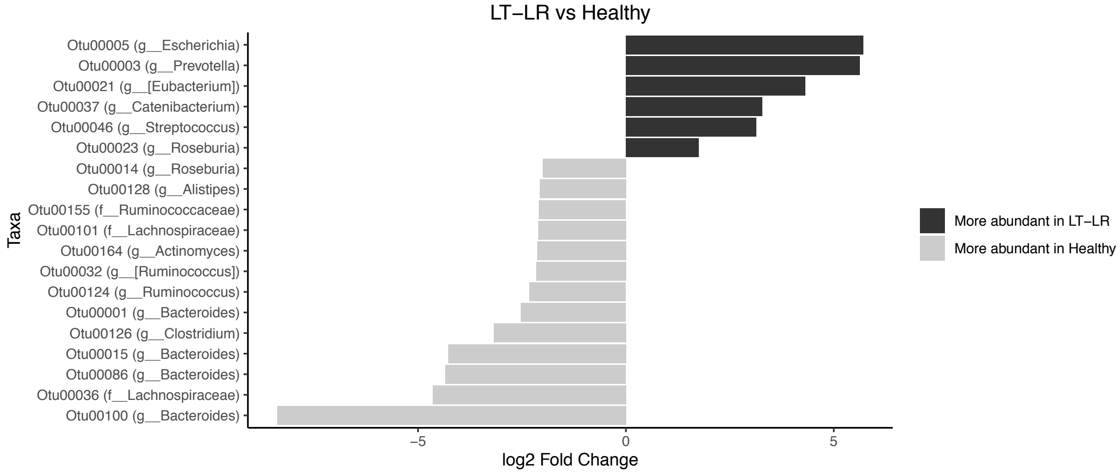
LT−LR vs Healthy
Otu00005 (g__Escherichia)
Otu00003 (g__Prevotella)
Otu00021 (g__[Eubacterium])
Otu00037 (g__Catenibacterium)
Otu00046 (g__Streptococcus)
Otu00023 (g__Roseburia)
Otu00014 (g__Roseburia)
Otu00128 (g__Alistipes)
Otu00155 (f__Ruminococcaceae)
Otu00101 (f__Lachnospiraceae)
Otu00164 (g__Actinomyces)
Otu00032 (g__[Ruminococcus])
Otu00124 (g__Ruminococcus)
Otu00001 (g__Bacteroides)
Otu00126 (g__Clostridium)
Otu00015 (g__Bacteroides)
Otu00086 (g__Bacteroides)
Otu00036 (f__Lachnospiraceae)
Otu00100 (g__Bacteroides)
Taxa
−5
0
5
log2 Fold Change
More abundant in LT−LR
More abundant in Healthy

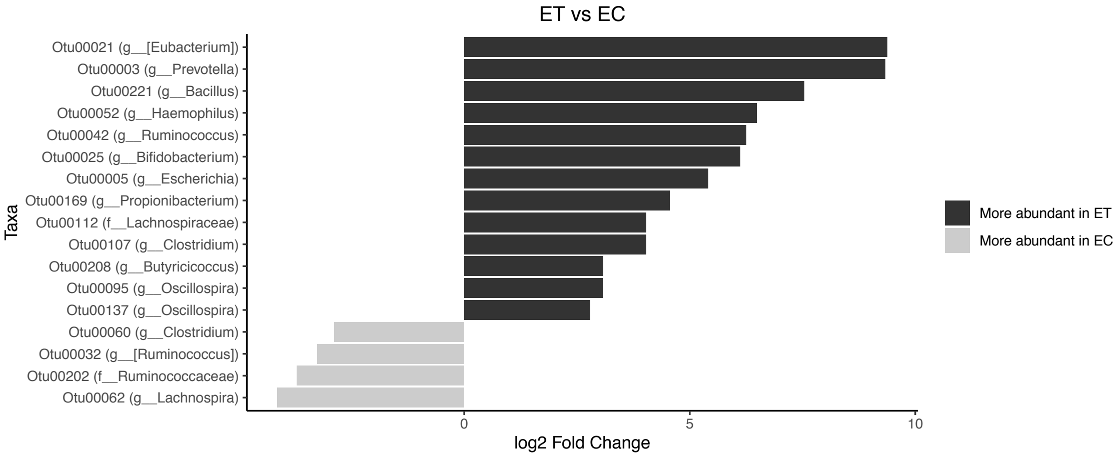

ET vs EC
Otu00021 (g__[Eubacterium])
Otu00003 (g__Prevotella)
Otu00221 (g__Bacillus)
Otu00052 (g__Haemophilus)
Otu00042 (g__Ruminococcus)
Otu00025 (g__Bifidobacterium)
Otu00005 (g__Escherichia)
Otu00169 (g__Propionibacterium)
Otu00112 (f__Lachnospiraceae)
Otu00107 (g__Clostridium)
Otu00208 (g__Butyricicoccus)
Otu00095 (g__Oscillospira)
Otu00137 (g__Oscillospira)
Otu00060 (g__Clostridium)
Otu00032 (g__[Ruminococcus])
Otu00202 (f__Ruminococcaceae)
Otu00062 (g__Lachnospira)
Taxa
0
5
10
log2 Fold Change
More abundant in ET
More abundant in EC

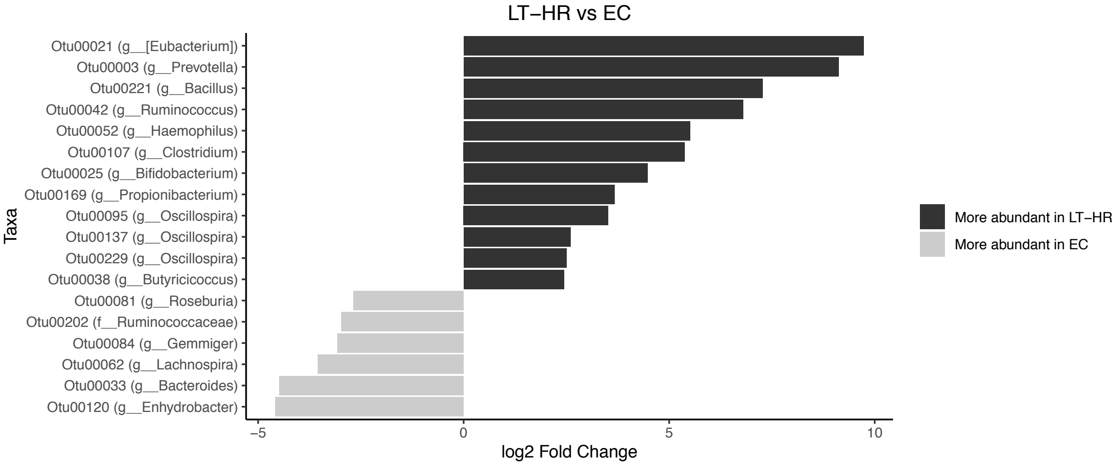

LT−HR vs EC
Otu00021 (g__[Eubacterium])
Otu00003 (g__Prevotella)
Otu00221 (g__Bacillus)
Otu00042 (g__Ruminococcus)
Otu00052 (g__Haemophilus)
Otu00107 (g__Clostridium)
Otu00025 (g__Bifidobacterium)
Otu00169 (g__Propionibacterium)
Otu00095 (g__Oscillospira)
Otu00137 (g__Oscillospira)
Otu00229 (g__Oscillospira)
Otu00038 (g__Butyricicoccus)
Otu00081 (g__Roseburia)
Otu00202 (f__Ruminococcaceae)
Otu00084 (g__Gemmiger)
Otu00062 (g__Lachnospira)
Otu00033 (g__Bacteroides)
Otu00120 (g__Enhydrobacter)
Taxa
−5
0
5
10
log2 Fold Change
More abundant in LT−HR
More abundant in EC

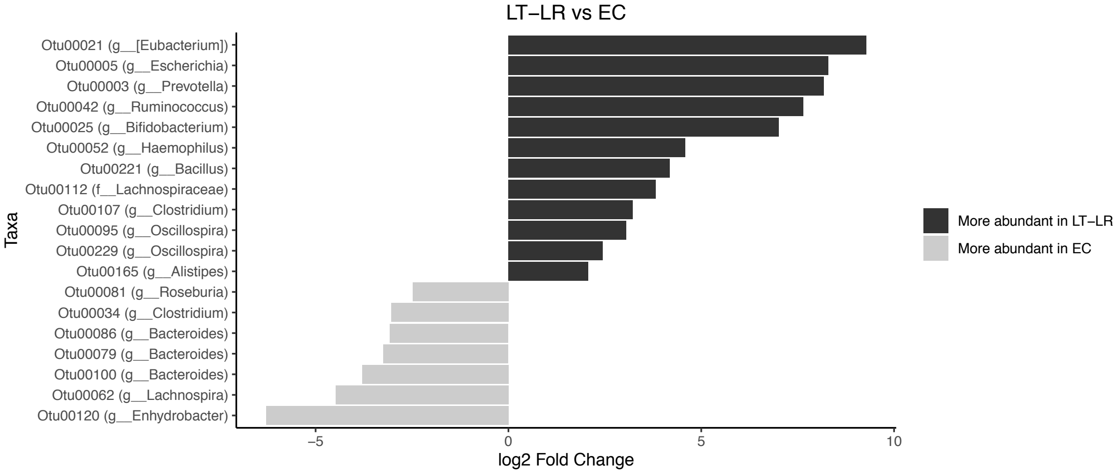

LT−LR vs EC
Otu00021 (g__[Eubacterium])
Otu00005 (g__Escherichia)
Otu00003 (g__Prevotella)
Otu00042 (g__Ruminococcus)
Otu00025 (g__Bifidobacterium)
Otu00052 (g__Haemophilus)
Otu00221 (g__Bacillus)
Otu00112 (f__Lachnospiraceae)
Otu00107 (g__Clostridium)
Otu00095 (g__Oscillospira)
Otu00229 (g__Oscillospira)
Otu00165 (g__Alistipes)
Otu00081 (g__Roseburia)
Otu00034 (g__Clostridium)
Otu00086 (g__Bacteroides)
Otu00079 (g__Bacteroides)
Otu00100 (g__Bacteroides)
Otu00062 (g__Lachnospira)
Otu00120 (g__Enhydrobacter)
Taxa
−5
0
5
10
log2 Fold Change
More abundant in LT−LR
More abundant in EC

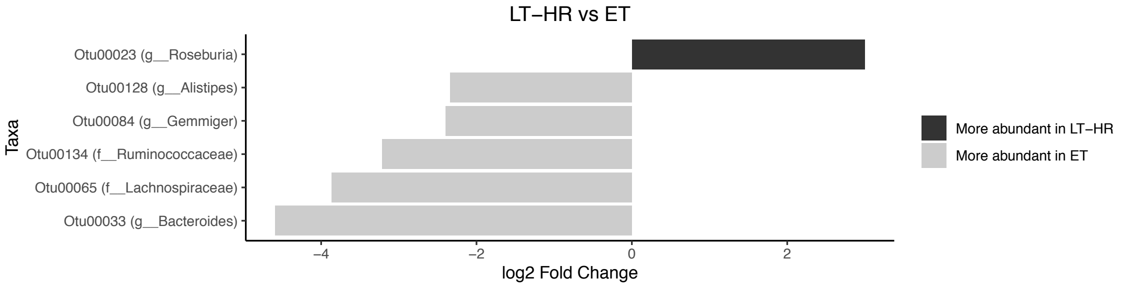
LT−HR vs ET
Otu00023 (g__Roseburia)
Otu00128 (g__Alistipes)
Otu00084 (g__Gemmiger)
Otu00134 (f__Ruminococcaceae)
Otu00065 (f__Lachnospiraceae)
Otu00033 (g__Bacteroides)
Taxa
−4
−2
0
2
log2 Fold Change
More abundant in LT−HR
More abundant in ET

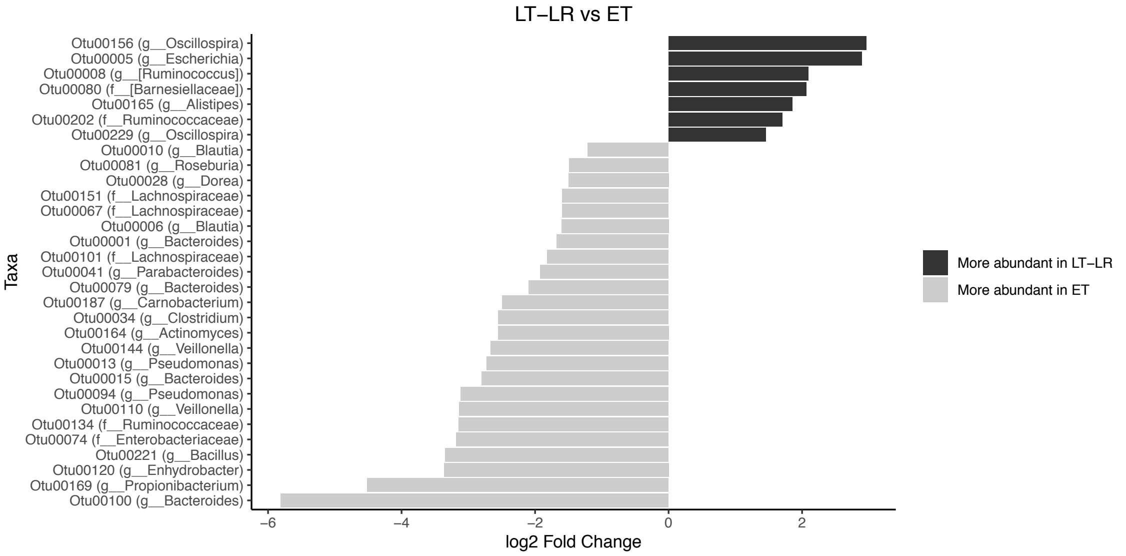
LT−LR vs ET
Otu00156 (g__Oscillospira)
Otu00005 (g__Escherichia)
Otu00008 (g__[Ruminococcus])
Otu00080 (f__[Barnesiellaceae])
Otu00165 (g__Alistipes)
Otu00202 (f__Ruminococcaceae)
Otu00229 (g__Oscillospira)
Otu00010 (g__Blautia)
Otu00081 (g__Roseburia)
Otu00028 (g__Dorea)
Otu00151 (f__Lachnospiraceae)
Otu00067 (f__Lachnospiraceae)
Otu00006 (g__Blautia)
Otu00001 (g__Bacteroides)
Otu00101 (f__Lachnospiraceae)
Otu00041 (g__Parabacteroides)
Otu00079 (g__Bacteroides)
Otu00187 (g__Carnobacterium)
Otu00034 (g__Clostridium)
Otu00164 (g__Actinomyces)
Otu00144 (g__Veillonella)
Otu00013 (g__Pseudomonas)
Otu00015 (g__Bacteroides)
Otu00094 (g__Pseudomonas)
Otu00110 (g__Veillonella)
Otu00134 (f__Ruminococcaceae)
Otu00074 (f__Enterobacteriaceae)
Otu00221 (g__Bacillus)
Otu00120 (g__Enhydrobacter)
Otu00169 (g__Propionibacterium)
Otu00100 (g__Bacteroides)
Taxa
−6
−4
−2
0
2
log2 Fold Change
More abundant in LT−LR
More abundant in ET

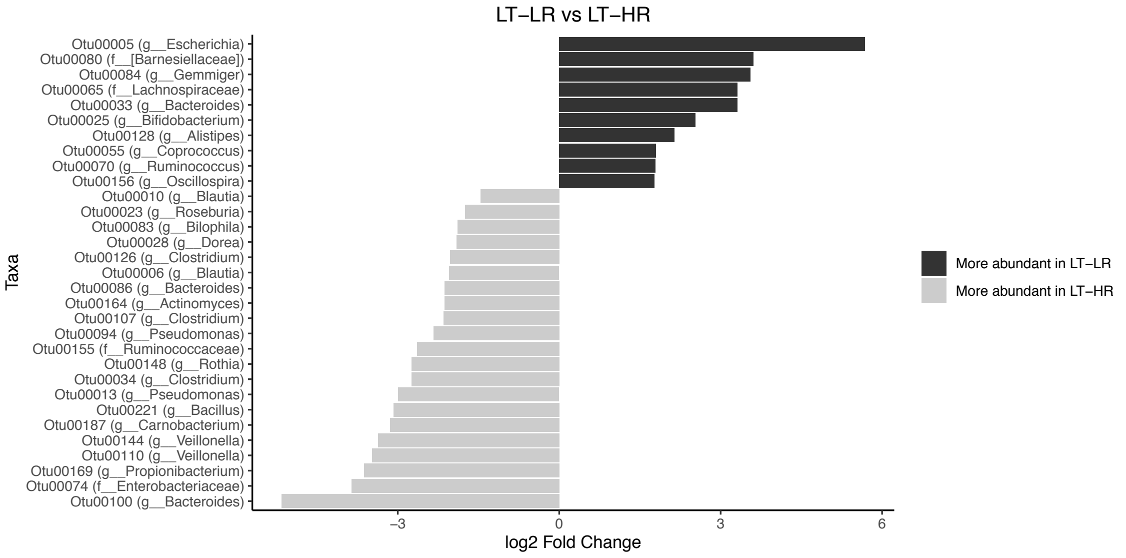
LT−LR vs LT−HR
Otu00005 (g__Escherichia)
Otu00080 (f__[Barnesiellaceae])
Otu00084 (g__Gemmiger)
Otu00065 (f__Lachnospiraceae)
Otu00033 (g__Bacteroides)
Otu00025 (g__Bifidobacterium)
Otu00128 (g__Alistipes)
Otu00055 (g__Coprococcus)
Otu00070 (g__Ruminococcus)
Otu00156 (g__Oscillospira)
Otu00010 (g__Blautia)
Otu00023 (g__Roseburia)
Otu00083 (g__Bilophila)
Otu00028 (g__Dorea)
Otu00126 (g__Clostridium)
Otu00006 (g__Blautia)
Otu00086 (g__Bacteroides)
Otu00164 (g__Actinomyces)
Otu00107 (g__Clostridium)
Otu00094 (g__Pseudomonas)
Otu00155 (f__Ruminococcaceae)
Otu00148 (g__Rothia)
Otu00034 (g__Clostridium)
Otu00013 (g__Pseudomonas)
Otu00221 (g__Bacillus)
Otu00187 (g__Carnobacterium)
Otu00144 (g__Veillonella)
Otu00110 (g__Veillonella)
Otu00169 (g__Propionibacterium)
Otu00074 (f__Enterobacteriaceae)
Otu00100 (g__Bacteroides)
Taxa
−3
0
3
6
log2 Fold Change
More abundant in LT−LR
More abundant in LT−HR

B

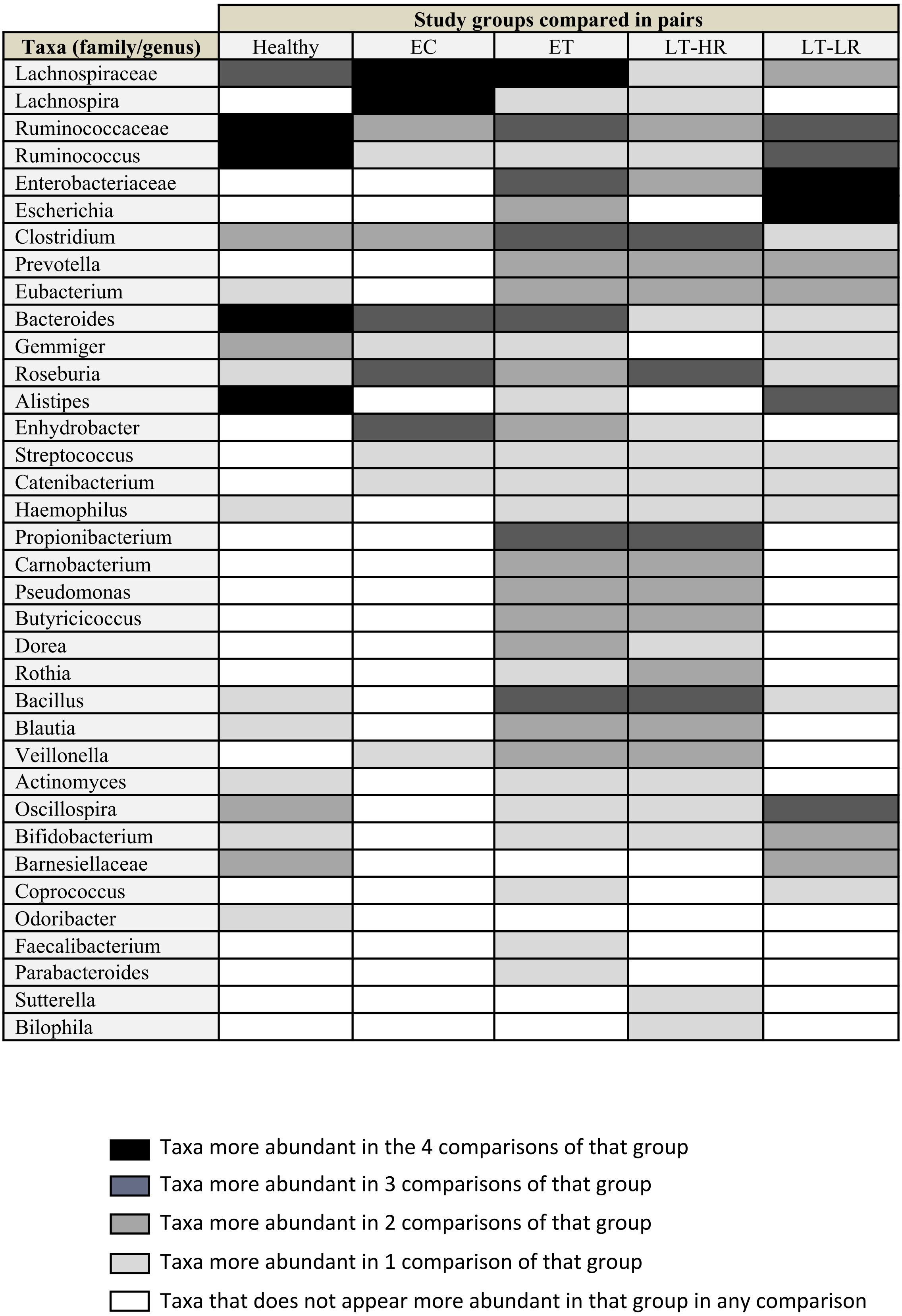
Study groups compared in pairs
Taxa (family/genus)
Healthy
EC
ET
LT-HR
LT-LR
Lachnospiraceae
Lachnospira
Ruminococcaceae
Ruminococcus
Enterobacteriaceae
Escherichia
Clostridium
Prevotella
Eubacterium
Bacteroides
Gemmiger
Roseburia
Alistipes
Enhydrobacter
Streptococcus
Catenibacterium
Haemophilus
Propionibacterium
Carnobacterium
Pseudomonas
Butyricicoccus
Dorea
Rothia
Bacillus
Blautia
Veillonella
Actinomyces
Oscillospira
Bifidobacterium
Barnesiellaceae
Coprococcus
Odoribacter
Faecalibacterium
Parabacteroides
Sutterella
Bilophila
Taxa more abundant in the 4 comparisons of that group
Taxa more abundant in 3 comparisons of that group
Taxa more abundant in 2 comparisons of that group
Taxa more abundant in 1 comparison of that group
Taxa that does not appear more abundant in that group in any comparison

Figure S4. A) Different OTUs obtained from gut mucosal samples showing significant differential relative abundance in comparisons established between different study groups (faced by pairs). B) Summary table calculated for a visual simplification of all comparisons (showed in S4A). Profiles of taxonomic grouping of more abundant OTUs, in their respective genus and/or family taxa, in comparisons of each study group with the rest of groups are shown. For example, Otu00062_g_Lachnospira would be included as Lachnospira genus but also as Lachnospiraceae family; however, Otu00112_f_Lachnospiraceae only includes Lachnospiraceae family and not Lachnospira genus, since it could include other genera of this family. As stated in the legend, intensity of grey in the squares indicates the number of comparisons in which the corresponding family or genus is more abundant in a specific study group. This is only a representation of data, without any statistical analysis. Note: f, family; g, genus.

Confirming the most differentially abundant OTUs present in the gut mucosa of different study groups by LEfSe analysis

DESeq2 results on OTUs abundance were corroborated in gut mucosal samples by an alternative method to calculate the effect size, LEfSe, which allowed to obtain linear discriminant analysis (LDA) scores for the five study groups when compared to each other (Fig. 3C). Thus, EC showed the highest LDA scores for Ruminococcus and Gemmiger (Ruminococcaceae), Clostridium and Dorea (Lachnospiraceae), as well as Bacteroides, Parabacteroides and Bilophila (Deltaproteobacteria; not in DESeq2 for this group). Healthy group, the one with more differentially abundant OTUs by LEfSe, presented in general great abundance of Bacteroidales order members (similar to EC), like Bacteroides, Alistipes and Odoribacter, as well as Clostridiales like Ruminococcus, Oscillospira, Blautia and Gemmiger. In ET group, Prevotella was the OTU with the highest LDA score, followed by Catenibacterium, Lachnospiraceae family and Bacillus. LT-HR mainly presented Actinobacteria (Bifidobacterium), Erysipelotrichi members and Eubacterium. LT-LR group showed a member of Coriobacteriia class, Collinsella, not present in DESeq2 analysis, as a highly abundant OTU, together with Clostridiales (Ruminococcus and Coprococcus). Taxonomic relationships of phylogeny among all

these OTUs obtained by LEfSe analysis were displayed using a cladogram (Fig. 3D). No additional information was obtained when abundance analyses were performed separating ileum and caecum samples (data not shown).

An OTUs-based signature from the gut-mucosal microbiome to distinguish HIV-subjects according to their immunological status upon cART

A random forest analysis of OTUs' relative abundances from gut-mucosal samples of treated HIV-groups (n=58) was performed to find out those OTUs whose relative abundance could contribute to classify treated-subjects based on their immune status. Initial analysis was performed with samples of the three treated HIV-groups separated, but a classification error of 73% with LT-HR samples, as many of them were assimilated as ET, and an Out-of-Bag error of 35%, uncovered the great overlap existing between ET and LT-HR groups. Once grouped together for further analyses, the Out-of-Bag error decreased to 12%. A subset of 30 OTUs classified samples as either ET/LT-HR or LT-LR, according to their relative abundances (Fig. 4A), with 100% of ET/LT-HR (40/40) and 61% of LT-LR (11/18) ileum and caecum samples being properly classified (error rate=12%). To improve discrimination, we chose the 14 most relevant OTUs (mean decrease accuracy, MDA>5) to build a multivariable logistic regression model that, after regularization, yielded 9 OTUs as the best candidates to predict belonging to ET/LT-HR or LT-LR groups (Fig. 4B): 5 Firmicutes (Blautia, Gemmiger, Clostridium, Ruminococcus and Oscillospira), 3 Bacteroidetes (Bacteroides, Parabacteroides and Barnesiellaceae) and 1 Proteobacteria (Escherichia). Using a ROC curve, predictions of this OTUs-based model gave an Area Under Curve (AUC) of 0.97.

Altered gut homeostasis, inflammation and immune activation parameters in late treated-subjects, especially in non-recoverers

Some peripheral (Fig. S5, panel A) and gut mucosa-related (Fig. S5, panel B) parameters were compared in subgroups of subjects belonging to the different treated-groups with available samples. Regarding soluble inflammation markers, significant lower levels of ß2-microglobulin were observed in ET compared to LT-LR ($p=0.02$) group, as well as less hsCRP in ET and LT-LR groups with respect to LT-HR ($p=0.006$ and $p=0.03$, respectively). A tendency towards higher levels of microbial translocation (LBP) could be seen in LT-LR after comparison with LT-HR and, especially, ET groups. LT-LR also showed higher levels of activated CD4 (HLA-DR+ CD38+) than LT-HR, which were significant when compared with ET ($p=0.003$) group. Upon assignment of a histological inflammation score to gut biopsies of the three groups, LT-LR showed significantly higher values than ET ($p=0.002$ in ileum and $p<0.0001$ in caecum) and LT-HR ($p=0.002$ in ileum). Caspase-3 expression showed differences among groups in ileum ($P_{KW}=0.02$) but not in caecum, although in both locations LT-LR group had the greatest values. By contrast, ET group showed not significant higher levels of mucin either in ileum and caecum mucosa than late-treated subjects. Finally, probably due to the increased permeability of the intestinal barrier, greater levels of zonulin expression were observed in LT-LR group compared to ET and LT-HR groups, with significant differences only in ileum (LT-LR vs LT-HR; $p=0.03$).

A *Peripheral*

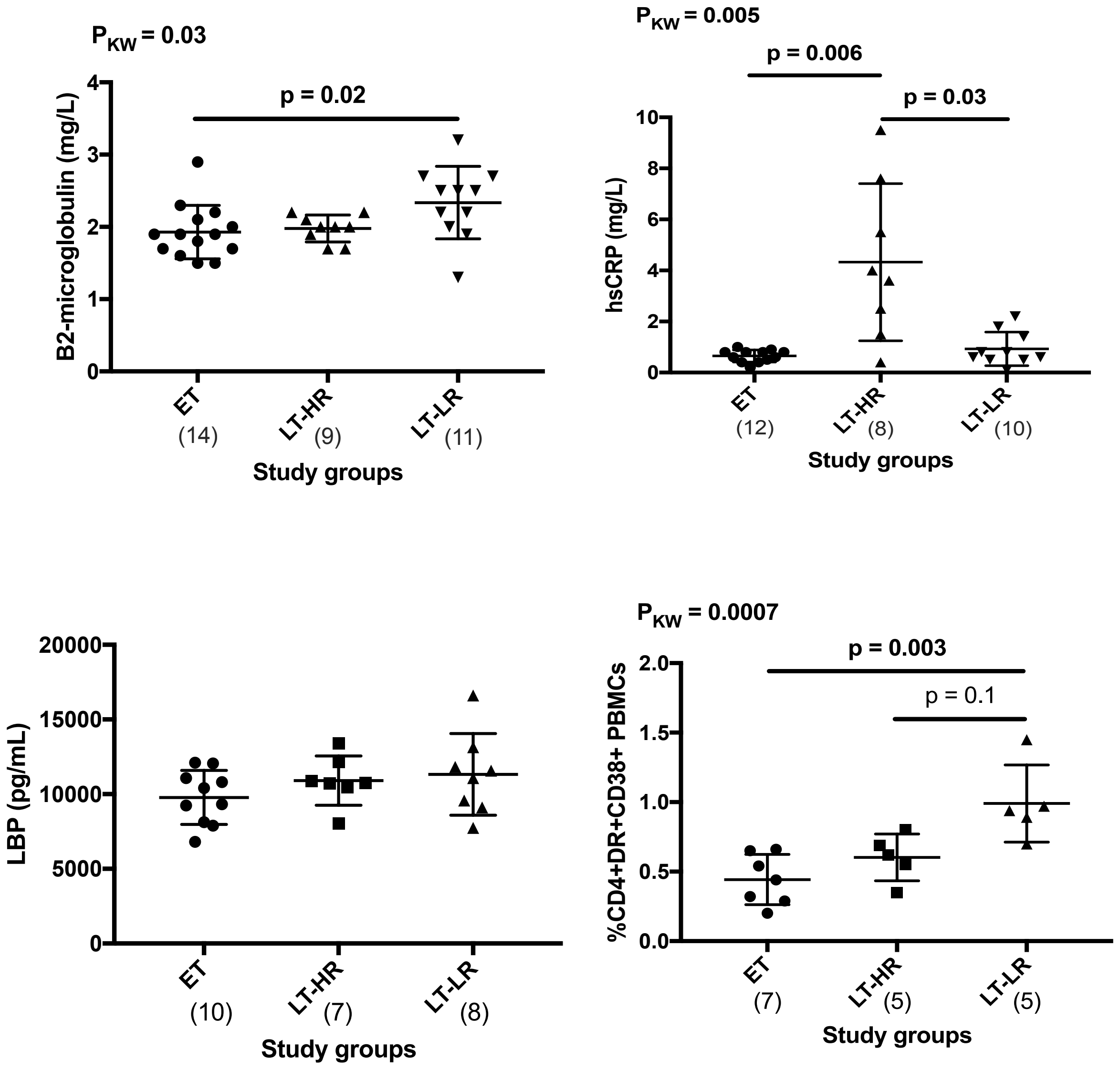

B *Gut mucosa-related*

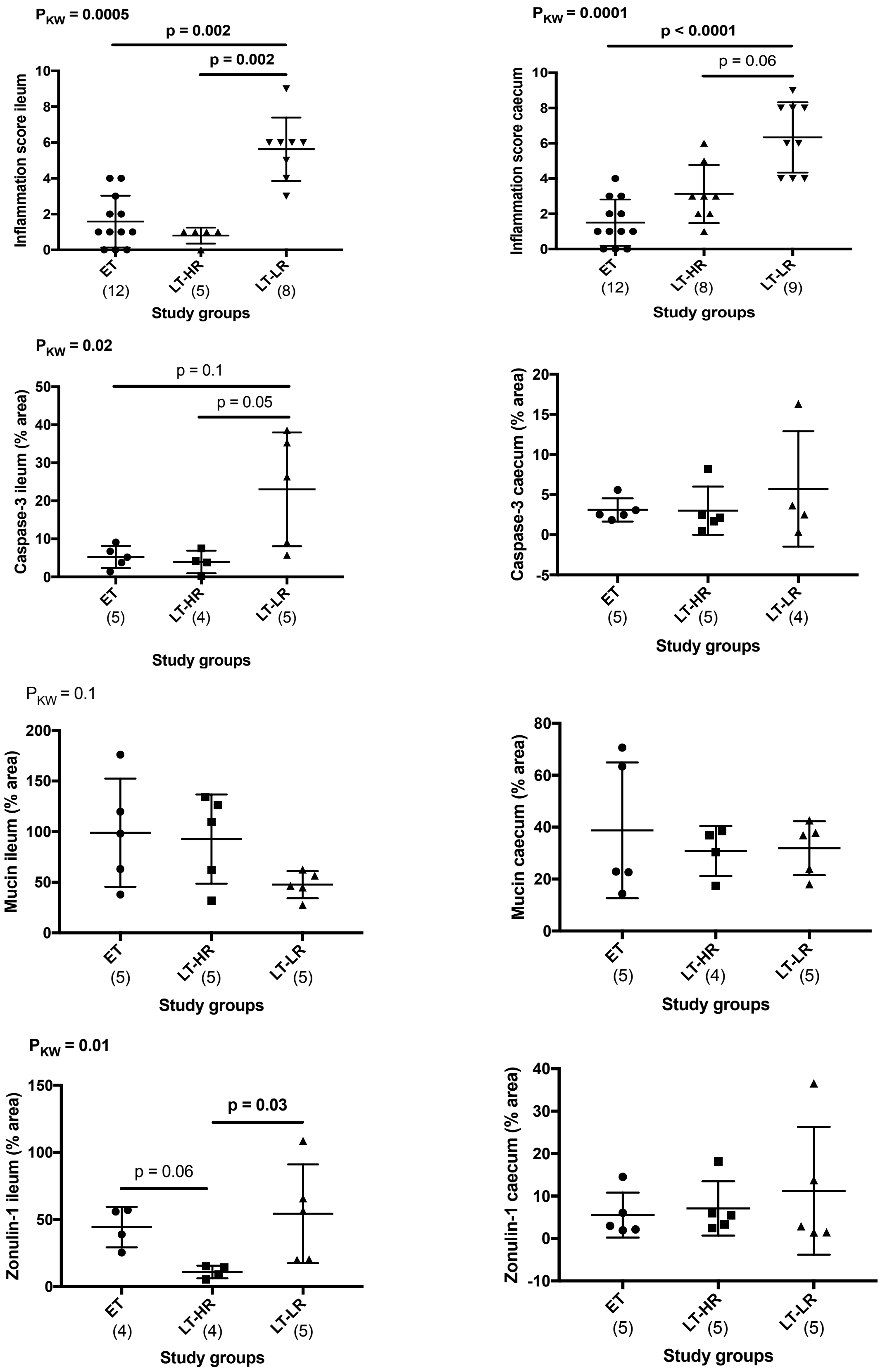

Figure S5. Significant differences found when different peripheral parameters of inflammation and immune activation (panel A) or gut mucosa-related markers of histopathological damage (panel B) were compared among cART-treated groups. Systemic inflammation markers were determined by serum immunoassays, whereas immune activation was analysed by immunophenotyping of PBMCs. Histopathological damage of the gut mucosa was assessed, by one hand, through a semi-quantitative score of severity (0-3) based on morphological findings affecting typical structures of the two anatomical sites studied (terminal ileum and caecum). In addition, proteins involved in the epithelial permeability of the gut mucosa were also evaluated by indirect immunofluorescence. Kruskal-Wallis and post-hoc Dunn's tests were used to estimate differences in blood and tissue parameters among different HIV-treated groups. $p<0.05$ indicates statistically significant differences. Numbers in brackets represent n of each group. Note: hsCRP: high-sensitivity C-reactive protein; LBP: LPS binding protein; PBMCs: peripheral blood mononuclear cells; % area: percentage of fluorescent area relative to Hoechst staining; P_{KW}: Kruskal Wallis' p value.

DISCUSSION

To the authors' knowledge, this is the first report to analyse the potential association of the immune status of HIV-subjects at cART onset with potential changes of composition and diversity of the gut microbiome. Furthermore, the mucosa of two different gut locations, terminal ileum and caecum, was analysed, as opposed to most of the studies based on fecal samples.[5, 28, 31] Late-treated-subjects recovering CD4 under cART (LT-HR) showed a similar dysbiotic profile in their gut mucosa to that of more immunopreserved early-treated subjects (ET). This was not the case of non-recoverers (LT-LR), despite displaying all late-treated subjects similar alpha-diversity values in their microbiota. Interestingly, in this work we also corroborate that (non-treated) elite controllers (EC) present a profile of abundant OTUs more typical of healthy subjects. In addition, a nine OTUs-based signature could be established for LT-LR, also affected by more inflammation, immune activation and gut tissue damage.

Our cohort of HIV-subjects represents one of the biggest used for the microbiome study, even with regard to the number of EC individuals included,[14] taking mucosal biopsies of different gut locations as the samples to be analysed.[12, 13, 17-20, 32, 33] Study groups showed age differences that might be behind some differences observed in the microbiome; however, a recent report covering all age groups found not significant dissimilarities in the gut microbiota of people within the same age range as subjects of our cohort (40-60 years), but in older people.[34] Study subjects were either not matched by sex given that gender does not seem to be a major cause of microbiome variation.[35] The worse immunological situation of LT-LR patients implied more previous HCV infections, as well as more time from diagnosis and under cART, concordant with others.[27, 36] Such poor immune status is reflected by the lower CD4 counts and CD4/CD8 ratio compared with rest of the groups.[5, 13, 24, 37, 38]

The absence of differences in alpha-diversity in our cohort is not a novelty.[5, 20, 27] Even though, the extended idea of the diversity loss in the intestinal microbiota upon HIV-infection[26] is reflected by a decreasing trend in alpha-diversity and richness values of treated-subjects when compared to healthy subjects. It is important to note that the significance of this finding is based on the majority of studies employing fecal samples, which normally show greater alpha-diversity values than gut biopsies,[20] probably due to the different microbiome composition and also by the marginal interactions with mucosal immune cells.[14, 39] Strickingly, among treated-subjects, non-recovery individuals presented higher alpha-diversity values than recoverers, but it has been also reported by others.[5, 27] Given that a higher percentage of low-recoverers received the drug combination NRTIs+INSTIs, a positive effect on diversity parameters cannot be completely ruled out.[40] Alternatively, a higher diversity in more progressed subjects (as LT-LR) could denote a worse microbiota profile, since it might imply the enrichment in harmful bacterial species that would be otherwise controlled in more immunopreserved scenarios. In this sense, the immunosenescent phenotype of low recoverers[41] may be contributing to this alpha-diversity increase as it appears to occur in elderly compared to younger cohorts.[42]

Only three previous studies compared the microbiome of different gut locations (all colon and ileum) in HIV-subjects, finding no differences in alpha-diversity between locations, being always greater in non-HIV than in HIV-subjects and without differences between responders and non-responders when those were analysed.[13, 19, 20] In agreement, we found a high correlation of different alpha-diversity measures between caecum and ileum in all groups' samples, so we can suppose that microbiome differences reported along the whole gut length[43] must be attenuated by the proximity of such anatomical sites. A possible sex-driven effect over gut microbiota diversity, possibly accounting in those

groups with more women (healthy and EC) showing higher values, was discarded when samples were grouped by sex, according also to recent findings in this sense.[44]

Following the line of many previous reports showing inter-individual differences between the gut bacterial community of non-HIV and HIV-subjects,[7, 23, 45] even in absence of cART,[46] our results on beta-diversity demonstrated a significant separation among healthy and treated-individuals. In terms of microbiome diversity, EC clearly clustered with healthy subjects.[24] Indeed, as a non-treated HIV-group, decreased diversity levels with respect to treated-ones could have been expected.[27, 40]

After comparing significantly abundant OTUs among study groups, one of the most important issues associated with HIV-infection was the increase of Proteobacteria in gut-mucosal samples of treated-subjects, to the point of reversing the normal Bacteroidetes/Proteobacteria ratio when all the differentially abundant OTUs were taken into account; being this one of the most common dysbiotic changes described both in untreated and treated-subjects, regardless the sample's nature.[4] At this phylum level, the gain of Firmicutes at the expense of Bacteroidetes seen in EC as compared to healthy group, as well as their loss of Actinobacteria respect to the rest of HIV-groups, was already reported with fecal samples.[8, 24] However, the presence of Actinobacteria in HIV-subjects has been controversial, presenting more abundance than in healthy controls in several fecal studies[24, 38] and less in others using gut-mucosal samples,[19] in contrast to our findings. On the other hand, it is reasonable that the greatest abundance of Bacteroidetes in healthy subjects could be related to a better preserved gut immune system and/or a non-dysbiotic state.

Despite being bacterial classes poorly mentioned in studies about gut microbiota in HIV-subjects, some previous findings, either in HIV or other inflammatory conditions, related Gammaproteobacteria and Erysipelotrichi, especially abundant in our treated-

subjects, to gut inflammatory damage.[45] Interestingly, Erysipelotrichi taxonomic members have been traditionally associated with HIV-infection[23] and even some reports defined them as possible markers of immune recovery.[27] In agreement, we found them more abundant in mucosal samples from ET and recoverers than in non-recoverers. By contrast, the decrease of Clostridia and Bacteroidia members in HIV-subjects was also reported in fecal studies comparing with healthy and EC subjects.[8, 47] Regarding the abundance of Bacilli in HIV-infected subjects, this bacterial class has been related to sexual preferences.[48]

Regarding family and genus taxa, Ruminococcaceae and Lachnospiraceae members were clearly less abundant in late-treated HIV-groups. As we show here, Ruminococcaceae abundance has been normally linked to a healthy condition both in fecal and gut-biopsy studies,[12, 30] and has also been positively correlated to CD4 counts.[30] Although the presence of Lachnospiraceae members in early-treated could be contradictory according to different results from mucosal[12, 13] and fecal[49] studies, its role in the gut environment is not still clear and was greatly abundant in HIV-subjects.[50] The increased abundance of Bacteroides and the depletion of Prevotella in healthy/EC subjects versus treated-subjects is one of the hallmarks of this kind of studies,[5] although likely biased towards the presence of homosexual subjects.[44] Eubacterium abundance observed in treated-groups contrasts with some previous findings,[40] but are in agreement with others showing a decreased abundance in healthy subjects.[9, 47] So, this alignment of bacterial OTUs between healthy and EC subjects may suggest that these subjects are able to keep a gut microbiota resembling a healthy environment.

Among treated-groups, as shown for the first time, ET and LT-HR subjects shared almost all their more abundant gut microbiome bacteria. Strickingly, either Propionibacterium and Butyricicoccus, only found in these two groups, and Roseburia

and Blautia, present also in healthy and EC, produce short-chain fatty acids (SCFAs) known to promote immunoregulatory and gut barrier functions.[4, 51] It is important to note that some of those OTUs appearing as abundant only in comparisons of ET/LT-HR groups could be eventually surrogate markers of immune preservation/recovery. On the other hand, LT-LR group showed an OTUs-abundance's pattern totally distinct from the rest, contrary to what reported Meyer-Myklestad et al. (2021),[20] who found no differences in microbiome composition of the gut mucosa between responders and non-responders. However, criteria for classification groups differed between this and our study. Interestingly, a dominant OTU in our non-recoverers was Escherichia, known proinflammatory pathobiont member of Enterobacteriaceae.[44] Coprococcus was already described in non-responders;[5] however, Barnesiellaceae, Oscillospira and Bifidobacterium were normally more associated to healthy states,[13, 27] though some of them were even found in HIV-infected subjects.[28, 45] The most important results observed in OTUs' abundance among gut mucosal samples of study groups were confirmed by an alternative LEfSe method. Therefore, it seems possible that cART instauration is able to partially restore the gut dysbiosis in recovering subjects, showing a profile of abundant OTUs very similar to that of a more immunopreserved early-treated group, but not in non-recoverers. Nevertheless, a role of the gut microbiome in the recovery of CD4 levels or the presence of a more resilient microbiota in subjects who finally recover their CD4 levels could not be discarded.

The similarity displayed in the gut microbiome of early-treated and recovering subjects was deeply studied by means of random forest and logistic regression analyses. Similar approaches were already used with fecal samples to explore bacterial gene richness or distinguish OTUs' signatures for men who have sex with men (MSM) and HIV-infection status.[26, 31, 44] However, to the authors' knowledge, this is the first model

to analyse OTUs' signatures in gut-mucosal samples of treated-subjects according to CD4 recovery levels. Even though several authors have associated some luminal OTUs to an immune recovery status,[7, 27] our nine gut-mucosal OTUs-based model was able to predict belonging to the non-recovery group with minimal error (5%) and area under curve of 0.97, regardless of gut location (ileum or caecum). Interestingly, and in line with our previous results, increases in Escherichia (potentially pathogenic genus) abundance and decreases in Bacteroides (linked to healthy gut environment) abundance would increase the probability of being classified as non-recoverer (LT-LR).

The close interactions established in the balance between gut microbiome and mucosal immune system could imply a more impaired immunological and systemic situation in late-treated low-recovery subjects as a reflection of their greater dysbiosis. Thus, comparison of different immune, biochemical and histological parameters among treated-groups mainly displayed a clear ladder-like pattern where non-recoverers showed higher levels of peripheral and tissue inflammation parameters (except for hsCRP), as well as bacterial translocation and immune activation than ET and, to a lesser extent, recovery subjects. These results agree with previous data on immune parameters compared between responders and non-responders.[36, 52] The greater damage to the gut epithelial integrity in LT-LR subjects was previously reported in similar studies using biopsy samples.[53, 54] Interestingly, there were no significant differences, in general, between ET and LT-HR groups in these analyses, concordant with microbiome results. To authors' knowledge, this is one of the few studies analysing presence of histologic damage in the gut mucosa of treated-subjects depending on their immune status.

Regarding the clinical utility of our findings, reversing gut dysbiosis and thus potentially inhibiting immune activation could be a strategy for improving the immune reconstitution of HIV-infected individuals with a non-recovery phenotype. This is

relevant since the possibility of correcting gut dysbiosis is becoming possible in the clinical context; from interventions aimed at modifying diet and even life habits to, more specifically, new strategies based on promising initial studies with pre- and pro-biotics as well as fecal microbiota transplants (FMT). This last approach has become a clinical tool capable of improving gut damage and HIV-associated dysbiosis.[55]

This study has some limitations. First, we did not include a viremic untreated group. The introduction of an early-treated group instead, rarely included in previous studies, allowed us to go deeper into the relationships between treated-groups. Second, results of EC group should be taken and interpreted with caution because of its small number of participants. Even so, this small group appeared highly homogeneous and other reports focused on EC included similar number of subjects, behaving similarly homogeneous.[14, 24] Finally, although healthy and HIV-groups were not matched by age and sex, either previous reports' data[35] and our proper results showed lack of effect on microbiome diversity at least within our ages range. Possible confounding effects of MSM presence (not documented) cannot be discarded.

CONCLUSIONS

In conclusion, low CD4 levels at cART onset and their recovery afterwards seems to be associated with partial restoration of the dysbiosis produced by HIV-infection in the gut mucosa of recoverers, but not in non-recovering subjects. Differentially abundant OTUs depict a profile of a non-recovery immune situation that was also related to parameters of inflammation, immune activation and gut histopathological damage. Prospective studies and experimental approaches would be needed to explore whether this OTUs-based signature is already present before cART onset.

ABBREVIATIONS

cART: combined antiretroviral therapy

HIV: human immunodeficiency virus

ET: early-treated

LT-HR: late-treated high recovery

LT-LR: late-treated low recovery

EC: elite controllers

OTU: operational taxonomic unit

GALT: gut-associated lymphoid tissue

HCV: hepatitis C virus

qPCR: quantitative polimerase chain reaction

LBP: lipopolysaccharide binding protein

ELISA: enzyme linked immunosorbent assay

hsCRP: high sensitivity C reactive protein

PBMCs: peripheral blood mononuclear cells

IQR: interquartile ranges

NMDS: non-metric multidimensional scaling

NRTI: nucleoside reverse transcriptase inhibitors

PI: protease inhibitors

INSTI: integrase strand transfer inhibitors

LDA: linear discriminant analysis

MDA: mean decrease accuracy

AUC: area under curve

SCFAs: short chain fatty acids

MSM: men who have sex with men

REFERENCES

1. Gori A, Tincati C, Rizzardini G, et al. Early impairment of gut function and gut flora supporting a role for alteration of gastrointestinal mucosa in human immunodeficiency virus pathogenesis. *J Clin Microbiol*. 2008;46:757-8.

2. Ellis CL, Ma ZM, Mann SK, et al. Molecular characterization of stool microbiota in HIV-subjects by panbacterial and order-level 16S ribosomal DNA (rDNA) quantification and correlations with immune activation. *J Acquir Immune Defic Syndr*. 2011;57:363-70.

3. Ceccarelli G, Statzu M, Santinelli L, et al. Challenges in the management of HIV-infection: update on the role of probiotic supplementation as a possible complementary therapeutic strategy for cART treated people living with HIV/AIDS. *Expert Opin Biol Ther*. 2019;19:949-65.

4. Vujkovic-Cvijin I, Somsouk M. HIV and the gut microbiota: composition, consequences, and avenues for amelioration. *Curr HIV/AIDS Rep*. 2019;16:204-13.

5. Lu W, Feng Y, Jing F, et al. Association between gut microbiota and CD4 recovery in HIV-1 infected patients. *Front Microbiol*. 2018;9:1451.

6. Armstrong AJS, Shaffer M, Nusbacher NM, et al. An exploration of Prevotella-rich microbiomes in HIV and men who have sex with men. *Microbiome*. 2018;6:198.

7. Lee SC, Chua LL, Yap SH, et al. Enrichment of gut-derived Fusobacterium is associated with suboptimal immune recovery in HIV-infected individuals. *Sci Rep*. 2018;8:14277.

8. Vesterbacka J, Rivera J, Noyan K, et al. Richer gut microbiota with distinct metabolic profile in HIV infected Elite Controllers. *Sci Rep*. 2017;7:6269.

9. Vázquez-Castellanos JF, Serrano-Villar S, Latorre A, et al. Altered metabolism of gut microbiota contributes to chronic immune activation in HIV-infected individuals. *Mucosal Immunol*. 2015;8:760-72.

10. Qing Y, Xie H, Su C, et al. Gut microbiome, short-chain fatty acids, and mucosa injury in young adults with human immunodeficiency virus infection. *Dig Dis Sci*. 2019;64:1830-43.

11. Bandera A, De Benedetto I, Bozzi G, Gori A. Altered gut microbiome composition in HIV-infection: causes, effects and potential intervention. *Curr Opin HIV AIDS*. 2018;13:73-80.

12. Dillon SM, Lee EJ, Kotter CV, et al. An altered intestinal mucosal microbiome in HIV-1 infection is associated with mucosal and systemic immune activation and endotoxemia. *Mucosal Immunol*. 2014;7:983-94.

13. Mutlu EA, Keshavarzian A, Losurdo J, et al. A compositional look at the human gastrointestinal microbiome and immune activation parameters in HIV infected subjects. *PLoS Pathog*. 2014;10:e1003829.

14. Vujkovic-Cvijin I, Dunham RM, Iwai S, et al. Dysbiosis of the gut microbiota is associated with HIV disease progression and tryptophan catabolism. *Sci Transl Med*. 2013;5:193ra91.

15. D'Angelo C, Reale M, Costantini E. Microbiota and probiotics in health and HIV-infection. *Nutrients*. 2017;9:615.

16. Dillon SM, Castleman MJ, Frank DN, et al. Inflammatory colonic innate lymphoid cells are increased during untreated HIV-1 infection and associated with markers of gut dysbiosis and mucosal immune activation. *J Acquir Immune Defic Syndr*. 2017;76:431–7.

17. Paquin-Proulx D, Ching C, Vujkovic-Cvijin I, et al. Bacteroides are associated with GALT iNKT cell function and reduction of microbial translocation in HIV-1 infection. *Mucosal Immunol*. 2017;10:69-78.

18. Coleman SL, Neff CP, Li SX, et al. Can gut microbiota of men who have sex with men influence HIV transmission? *Gut Microbes*. 2020;11:610-9.

19. Gianella S, Chaillon A, Mutlu EA, et al. Effect of cytomegalovirus and Epstein-Barr virus replication on intestinal mucosal gene expression and microbiome composition of HIV-infected and uninfected individuals. *AIDS*. 2017;31:2059-67.

20. Meyer-Myklestad MH, Medhus AW, Lorvik KB, et al. HIV-infected immunological non-responders have colon-restricted gut-mucosal immune dysfunction. *J Infect Dis*. 2020;jiaa714.

21. The Late Presentation Working Groups in EuroSIDA and COHERE. Estimating the burden of HIV late presentation and its attributable morbidity and mortality across Europe 2010–2016. *BMC Infect Dis*. 2020;20:728.

22. Engsig FN, Zangerle R, Katsarou O, et al. Long-term mortality in HIV-positive individuals virally suppressed for >3 years with incomplete CD4 recovery. *Clin Infect Dis*. 2014;58:1312–21.

23. Lozupone CA, Li M, Campbell TB, et al. Alterations in the gut microbiota associated with HIV-1 infection. *Cell Host Microbe*. 2013;14:329-39.

24. Nowak P, Troseid M, Avershina E, et al. Gut microbiota diversity predicts immune status in HIV-1 infection. *AIDS*. 2015;29:2409-18.

25. Pinto-Cardoso S, Klatt NR, Reyes-Terán G. Impact of antiretroviral drugs on the microbiome: unknown answers to important questions. *Curr Opin HIV AIDS*. 2018;13:53-60.

26. Noguera-Julian M, Rocafort M, Guillén Y, et al. Gut microbiota linked to sexual preference and HIV-infection. *EBioMedicine*. 2016;5:135-46.

27. Serrano-Villar S, Rojo D, Martínez-Martínez M, et al. Gut bacteria metabolism impacts immune recovery in HIV-infected individuals. *EBioMedicine*. 2016;8:203-16.

28. Deusch S, Serrano-Villar S, Rojo D, et al. Effects of HIV, antiretroviral therapy and prebiotics on the active fraction of the gut microbiota. *AIDS*. 2018;32:1229-37.

29. Vázquez-Castellanos JF, Serrano-Villar S, Jiménez-Hernández N, et al. Interplay between gut microbiota metabolism and inflammation in HIV-infection. *ISME J*. 2018;12:1964-76.

30. Xie Y, Sun J, Wei L, et al. Altered gut microbiota correlate with different immune responses to HAART in HIV-infected individuals. *BMC Microbiol*. 2021;21:11.

31. Guillén Y, Noguera-Julian M, Rivera J, et al. Low nadir CD4+ T-cell counts predict gut dysbiosis in HIV-1 infection. *Mucosal Immunol*. 2019;12:232-46.

32. Dillon SM, Kibbie J, Lee EJ, et al. Low abundance of colonic butyrate-producing bacteria in HIV infection is associated with microbial translocation and immune activation. *AIDS*. 2017;31:511-21.

33. Kelley CF, Kraft CS, de Man TJB, et al. The rectal mucosa and condomless receptive anal intercourse in HIV-negative MSM: implications for HIV transmission and prevention. *Mucosal Immunol*. 2017;10:996-1007.

34. Xu C, Zhu H, Qiu P. Aging progression of human gut microbiota. *BMC Microbiol*. 2019;19:236.

35. Huttenhower C, Gevers D, Knight R, et al. (The Human Microbiome Project Consortium). Structure, function and diversity of the healthy human microbiome. *Nature*. 2013;486:207-14.

36. Serrano-Villar S, Vázquez-Castellanos JF, Vallejo A, et al. The effects of prebiotics on microbial dysbiosis, butyrate production and immunity in HIV-subjects. *Mucosal Immunol*. 2017;101279-93.

37. Ji Y, Zhang F, Zhang R, et al. Changes in intestinal microbiota in HIV-1-infected subjects following cART initiation: influence of CD4+ T cell count. *Emerg Microbes Infect*. 2018;7:113.

38. Serrano-Villar S, Sánchez-Carrillo S, Talavera-Rodríguez A, et al. Blood bacterial profiles associated with human immunodeficiency virus infection and immune recovery. *J Infect Dis*. 2021;223:471-81.

39. Zmora N, Zilberman-Schapira G, Suez J, et al. Personalized gut-mucosal colonization resistance to empiric probiotics is associated with unique host and microbiome features. *Cell*. 2018;174:1388-1405.

40. Villanueva-Millán M, Pérez-Matute P, Recio-Fernández E, Lezana Rosales J, Oteo J. Differential effects of antiretrovirals on microbial translocation and gut microbiota composition of HIV-infected patients. *J Int AIDS Soc*. 2017;20:21526.

41. Molina-Pinelo S, Vallejo A, Díaz L, et al. Premature immunosenescence in HIV-infected patients on highly active antiretroviral therapy with low-level CD4 T cell repopulation. *J Antimicrob Chemother*. 2009;64:579-88.

42. Ruiz-Ruiz S, Sánchez-Carrillo S, Ciordia S, et al. Functional microbiome deficits associated with ageing: Chronological age threshold. *Aging Cell*. 2020;19:e13063.

43. Thursby E, Juge N. Introduction to the human gut microbiota. *Biochem J*. 2017;474:1823-36.

44. Vujkovic-Cvijin I, Sortino O, Verheij E, et al. HIV-associated gut dysbiosis is independent of sexual practice and correlates with noncommunicable diseases. *Nat Commun*. 2020;11:2448.

45. Dinh DM, Volpe GE, Duffalo C, et al. Intestinal microbiota, microbial translocation, and systemic inflammation in chronic HIV-infection. *J Infect Dis*. 2015;211:19-27.

46. Sortino O, Phanuphak N, Schuetz A, et al. (RV254/SEARCH010 Study Group). Impact of acute HIV-infection and early antiretroviral therapy on the human gut microbiome. *Open Forum Infect Dis*. 2019;7:ofz367.

47. Rocafort M, Noguera-Julian M, Rivera J, et al. Evolution of the gut microbiome following acute HIV-1 infection. *Microbiome*. 2019;7:73.

48. Zhou Y, Ou Z, Tang X, et al. Alterations in the gut microbiota of patients with acquired immune deficiency syndrome. *J Cell Mol Med*. 2018;22:2263-71.

49. Kaur US, Shet A, Rajnala N, et al. High Abundance of genus Prevotella in the gut of perinatally HIV-infected children is associated with IP-10 levels despite therapy. *Sci Rep*. 2018;8:17679.

50. San-Juan-Vergara H, Zurek E, Ajami NJ, et al. A Lachnospiraceae-dominated bacterial signature in the fecal microbiota of HIV-infected individuals from Colombia, South America. *Sci Rep*. 2018;8:4479.

51. Kelly CJ, Zheng L, Campbell EL, et al. Crosstalk between microbiota-derived short-chain fatty acids and intestinal epithelial HIF augments tissue barrier function. *Cell Host Microbe*. 2015;17:662-71.

52. Malazogu F, Rousseau RK, Shivappa N, et al. The dietary inflammatory index is not associated with gut permeability or biomarkers of systemic inflammation in hiv immunologic non-responders. *Front Nutr*. 2021; 8:736816.

53. Somsouk M, Estes JD, Deleage C, et al. Gut epithelial barrier and systemic inflammation during chronic HIV-infection. *AIDS*. 2015;29:43-51.

54. Lorvik KB, Meyer-Myklestad MH, Kushekar K, et al. Enhanced gut-homing dynamics and pronounced exhaustion of mucosal and blood cd4+ t cells in hiv-infected immunological non-responders. *Front Immunol*. 2021; 12:744155.

55. Serrano-Villar S, Talavera-Rodríguez A, Gosalbes MJ, et al. Fecal microbiota transplantation in HIV: A pilot placebo-controlled study. *Nat Commun*. 2021;12:1139.

ANNEXES

- **Annex S1.** Complete R code used to do data analysis of the microbiota present in all the ileum and caecum mucosal biopsy samples from different study groups: **S1a.Rmd. (can be opened the same as a .txt file)** Report containing the R code to perform secondary/tertiary data analysis. **S1b.taxonomy. (can be opened the same as a .txt file)** Taxonomy file from mothur, storing the consensus taxonomy for each OTU. **S1c.shared. (can be opened the same as a .txt file)** Shared file from mothur, representing the number of times that an OTU is observed in multiple samples. **S1d.tsv. (can be opened the same as a .txt file)** samples metadata file with clinical/biological information of interest for each sample.

Due to the format and, especially, their long extension (more than 2,000 pages in total), these files cannot be added to the document. Nevertheless, they will be fully available upon reasonable request to the corresponding author.

- **Annex S2.** Age comparison between healthy and rest of the study subjects (HIV groups: EC, ET, LT-HR and LT-LR). Note: ns, non-significant.

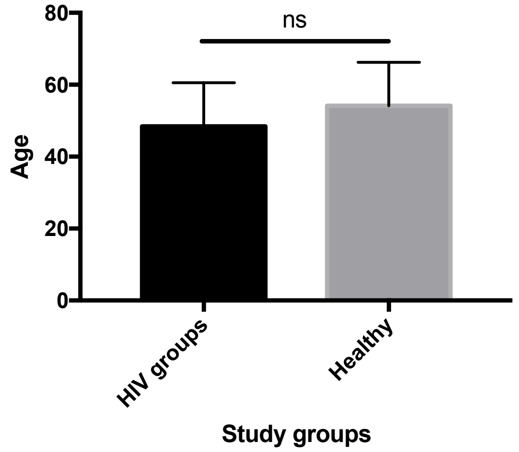

Supplement: Supplementary file 1 — Supporting information. [file CTM2-12-e788-s001.pdf]
